# Synthesis and Biological Evaluation of New Bis-Indolinone Derivatives Endowed with Cytotoxic Activity

**DOI:** 10.3390/molecules26206277

**Published:** 2021-10-16

**Authors:** Rita Morigi, Elena Catanzaro, Alessandra Locatelli, Cinzia Calcabrini, Valentina Pellicioni, Alberto Leoni, Carmela Fimognari

**Affiliations:** 1 Department of Pharmacy and Biotechnology, Alma Mater Studiorum—University of Bologna, Via Belmeloro 6, 40126 Bologna, Italy; rita.morigi@unibo.it (R.M.); alberto.leoni@unibo.it (A.L.); 2 Department for Life Quality Studies, Alma Mater Studiorum—University of Bologna, Corso d’Augusto 237, 47921 Rimini, Italy; elena.catanzaro2@unibo.it (E.C.); ccalcabrini@hotmail.com (C.C.); valentina.pellicion2@unibo.it (V.P.); carmela.fimognari@unibo.it (C.F.)

**Keywords:** programmed cell death, cell cycle, small molecules, indolinone systems, Knoevenagel reaction

## Abstract

A series of new Knoevenagel adducts, bearing two indolinone systems, has been synthesized and evaluated on 60 human cancer cell lines according to protocols available at the National Cancer Institute (Bethesda, MD, USA). Some derivatives proved to be potent antiproliferative agents, showing GI_50_ values in the submicromolar range. Compound **5b** emerged as the most active and was further studied in Jurkat cells in order to determine the effects on cell-cycle phases and the kind of cell death induced. Finally, oxidative stress and DNA damage induced by compound **5b** were also analyzed.

## 1. Introduction

Noncommunicable diseases (NCDs) are now responsible for the majority of global deaths, and cancer is expected to rank as the leading cause of death and the single most important barrier to increasing life expectancy in every country of the world in the 21st century [1]. Despite a variety of agents already used in anticancer therapy, drug resistance, toxicity, and side effects remain problems to be solved; hence, the development of new molecules still represents an attractive objective.

Programmed cell death (PCD) is a collective name that indicates any form of cellular death governed by an intracellular program, such as apoptosis, necroptosis, and ferroptosis. As opposed to accidental necrosis, PCDs allow cell death, maintaining cellular and tissue homeostasis and, in some instances, triggering a full-blown immune response [2,3,4]. For a long time, apoptosis has been considered the only PCD, but it has been clear from the last 60 years that cells can also commit suicide in a caspase-independent way. For instance, necroptosis is a form of regulated necrosis mediated by receptor-interacting protein kinase 1 (RIPK1), RIPK3, and pseudokinase mixed lineage kinase domain-like (MLKL). It has been discovered as an alternative regulated pathway triggered when apoptosis is impaired, and now, it is clear that it also represents a promising therapeutic target for chemotherapy [5]. Ferroptosis, on its side, is an iron-dependent PCD characterized by the accumulation of lipid peroxides. It was characterized only in 2012 but already represents an appealing alternative to necroptosis and apoptosis to overcome the unsatisfactory efficacy of current therapies [6,7]. Collectively, inducing one or more than one PCD at the same time accounts for an effective way to increase the chance of a successful therapeutic outcome.

**Scheme 1:**
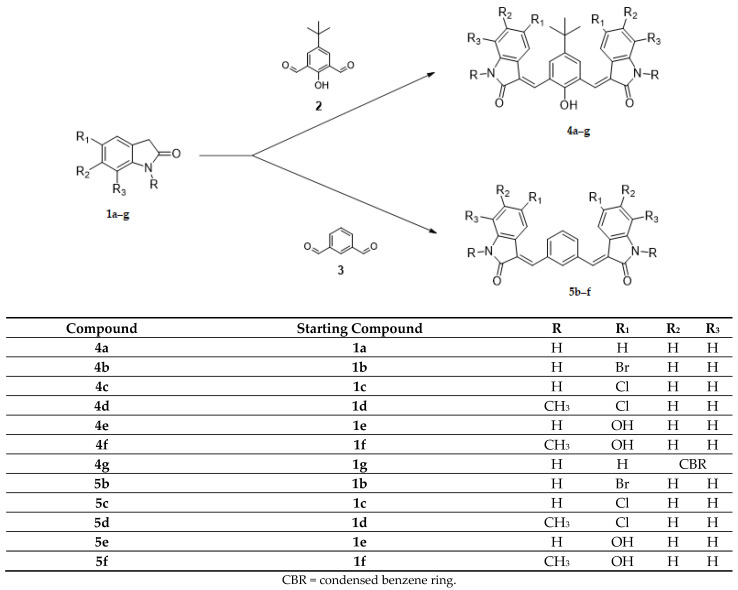
Synthesis of new derivatives **4a–g**, **5b–f**.

**Scheme 2:**
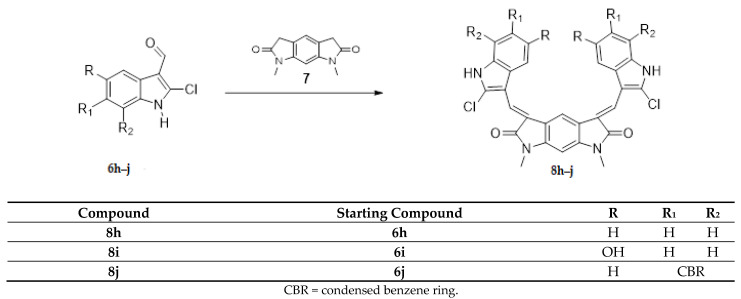
Synthesis of new derivatives **8h–j**.

The 2-indolinone scaffold holds a pivotal place as a pharmacophore for the development of anticancer agents, and, in addition, this scaffold has many pharmacological activities [8]. In this field, our research group published many studies concerning new 2-indolinone derivatives as antiproliferative agents [9,10,11,12]. Some of our previous studies were devoted to the synthesis of bis-indole derivatives formed by two indole systems separated by a central moiety. Through this strategy, it was possible to identify several compounds endowed with a marked inhibition of cellular proliferation. Based on the most active antitumor agents previously described [13,14], this paper reports the synthesis, the studies on antitumor activity and mechanism of action of new derivatives with a framework bearing two indolinone nucleus or a central core pyrroloindoledione. The new derivatives were designed with the following rationale:

(1) the core, tetra-substituted benzene (**4a–g**) and di-substituted benzene (**5b–f**), has been maintained, and new substituents in the indolinone systems have been introduced (Scheme 1);

(2) the core has been substituted with 1,7-dimethyl-5,7-dihydropyrrolo [3,2-f]indole-2,6(1H,3H)-dione while maintaining the indole wings with different substituents (Scheme 2, compounds **8h–j**).

In both cases, 2-indolinone is the scaffold of the new derivatives.

The antitumor activity of all the new compounds was evaluated according to the protocols available at the National Cancer Institute, Bethesda, MD (NCI).

In addition, we examined the efficacy of the most active compound to induce PCD on Jurkat cells.

## 2. Results and Discussion

### 2.1. Chemistry

The Knoevenagel reaction, employed in the synthesis of the new derivatives, involved the condensation of a carbonyl group with a methylene activated by a neighboring electron withdrawing group (Scheme 1 and Scheme 2). In the first case (Scheme 1, compounds **4a–g**; **5b–f**), the carbonyl groups were in the core, whereas the active methylene groups were in the indolinone wings. In the second case (Scheme 2, compounds **8h–j**), this situation was inverted: the active methylene groups were in the core (pyrrolo [3,2-f]indole-2,6(1H,3H)-dione ring), whereas the carbonyl groups were in the indole wings.

Most of the new derivatives have been prepared with the same procedure described for the previously published compounds [13,14]: the appropriate indolinone **1** or **7** in methanol has been treated with the appropriate dicarbaldehyde **2**,**3** or **6** in the presence of piperidine (method 1). Since not all the designed compounds have been obtained under these reaction conditions, for the synthesis of compounds **4d–e**, ethanol and HCl conc. have been used (method 2); for compound **5b**, a mixture of acetic acid/hydrochloric acid has been employed (method 3); while for compounds **5c–d**, toluene and p-toluenesulfonic acid have been used (method 4).

The structures of the final compounds were confirmed by means of IR, ^1^H-NMR, ^13^C-NMR, and HRMS spectra. All of them were obtained as almost pure geometrical isomers, but their stability in DMSO-d_6_ is not the same for all the compounds.

According to the ^1^H-NMR spectra, all the compounds were obtained as *E* isomers, except **4e**, **5b**–**e**, and **8j**, which also contain a small amount of the *Z* isomer. Furthermore, the long ^13^C-NMR acquisition times required, due to the presence of numerous quaternary carbons, led to complex mixtures as in the case of compound **5b** (see Supporting Information). Therefore, for these compounds (**4e**, **5c**–**d**, **f**) the ^13^C-NMR spectra were not recorded as the isomerization increases in DMSO-d_6_ solution. The geometrical configuration was determined by performing NOE (Nuclear Overhauser Effect) experiments on derivatives **4b** and **8h** in order to evaluate whether the methine bridge and the proton at the 4 position of the indole (ind-4) are close in space (Z configuration) or not (E configuration). First of all, we studied compound **4b**, and we noticed that the geometrical configuration of the two centers is the same, since the couple of NH groups and the other couples of indole protons (ind-4, ind-6, ind-7) give a single signal. The irradiation of ind-4 (7.58 ppm) produced NOE at the -C(CH_3_)_3_ group (singlet 1.33 ppm) and at the aromatic protons (singlet 7.77 ppm), whereas NOE was not observed at the –CH = proton; the irradiation of the singlet at 1.33 ppm -(C(CH_3_)_3_) produced NOE at 7.77 (aromatic protons) and at 7.58 ppm (ind-4). These data are in agreement with the *E* configuration.

The spectrum of derivative **8h** also shows that the geometrical configuration of the two centers is the same. For this compound, the irradiation of –CH= protons at 7.37 ppm did not produce any effect on other protons. The lack of NOE demonstrates that even this compound belongs to the *E* configuration.

As far as compounds **5** are concerned, since the peaks of the ^1^H-NMR spectra were too near for significant NOE experiments, we compared the common features of analog derivatives [14]. On this basis, we believe that these compounds also have the *E* configuration.

### 2.2. Biological Studies

#### 2.2.1. Effects in Cultured Human Tumor Cell Lines

As a primary screening, the new compounds were submitted to the Developmental Therapeutics Program (DTP) at the National Cancer Institute (NCI) (http://dtp.nci.nih.gov) for evaluation of antitumor activity in the human cell line screen. In the preliminary test, compounds were tested at a single high concentration (10 µM) in the full NCI 60 cell panel (NCI 60 Cell One-Concentration Screen). This panel is organized into subpanels representing leukemia, melanoma, and cancers of the lung, colon, kidney, ovary, breast, prostate, and central nervous system. Only compounds with predetermined threshold inhibition criteria in a minimum number of cell lines progress to the full 5-concentration assay. These criteria were selected to efficiently capture compounds with antiproliferative activity based on careful analysis of historical DTP screening data. The results are expressed as the percentage of growth of treated cells relative to the control following a 48-h incubation. The one-concentration data is a mean graph of the percent growth of treated cells (unpublished results).

All but three of the compounds tested were subjected to the full 5-concentration assay. They were dissolved in DMSO and evaluated using five concentrations at ten-fold dilutions, the highest being 100 µM. Table 1 shows the results obtained (vincristine is reported for comparison purposes), which are expressed at three assay endpoints: the 50% growth inhibitory power (GI_50_), the cytostatic effect (TGI = Total Growth Inhibition), and the cytotoxic effect (LC_50_). For some derivatives, the 5-concentration test was repeated and no significant differences were found; in this case, the data reported in Table 1 are the mean values between the two experiments.

#### 2.2.2. Structure–Activity Relationships

(a) Wings Modification

As far as the 4-*tert*-butyl-2,6-diformylphenol core is concerned, the shift of the chlorine from position 4 to 5 of the indolinone system increased the activity of compound **4c** (mean GI_50_ = 1.15 μM), which was more active than its parent compound described in the previous paper: (mean GI_50_ = 2.7 μM) [14], even in case of introduction of a methyl group in the indolinone NH (**4d,** mean GI_50_ = 2.04 μM), whereas the substitution of the chlorine with a bromine decreased the activity (**4b,** mean GI_50_ = 2.88 μM). The introduction of a hydroxy group in the same position led to a loss of activity (compound **4e** does not progress to the full five-concentrations assay), whereas the simultaneous introduction of a methyl group in the indolinone NH maintained the efficacy (**4f**, mean GI_50_ = 3.09 μM). A marked decrease in activity was observed both in the absence of substituents on the indolinone (**4a**, mean GI_50_ = 5.13 μM), as well as by introducing a condensed benzene ring (**4g**, mean GI_50_ = 7.76 μM). The most active derivatives (**4b–d**) were more effective toward the CNS tumor cell lines (GI_50_ 2.19; 1.29; 1.78 µM respectively).

When the core is a benzene ring, the shift of the chlorine from position 4 to 5 of the indolinone system led also to activity improvement of derivative **5c** (mean GI_50_ = 1.15 μM), which proved to be more active than its parent compound described in the previous paper [14] (mean GI_50_ = 2.4 μM), even in the case of introduction of a methyl group in the NH indolinone (**5d**, mean GI_50_ = 2.04 μM). A particular mention is due to the substitution of the Cl with Br in position 5, which led to the most active compound of the whole series (**5b**, mean GI_50_ = 0.91 μM). The introduction of a hydroxy group in the same position was detrimental, whereas the simultaneous introduction of a methyl group in the NH indolinone increased the activity (**5f**, mean GI_50_ = 1.82 μM). All these derivatives were subjected to the full 5-concentration assay; **5b–d** and **f** were especially active toward leukemia cell lines (GI_50_ 0.51; 0.56; 0.55; 0.87 µM, respectively).

Considering the potency and toxicity on leukemia cell lines, it is important to note that both series of compounds **4** and **5** showed a great difference between GI_50_ and LC_50_.

(b) Core modification

The substitution of the core with 1,7-dimethyl-5,7-dihydropyrrolo[3,2-f]indole-2,6(1H,3H)-dione (**8h–j**) led to a loss of activity; only one of the three compounds tested (**8h**) was subjected to the full 5-concentration assay.

#### 2.2.3. Effect on Jurkat Cells Proliferation

**Figure 1:**
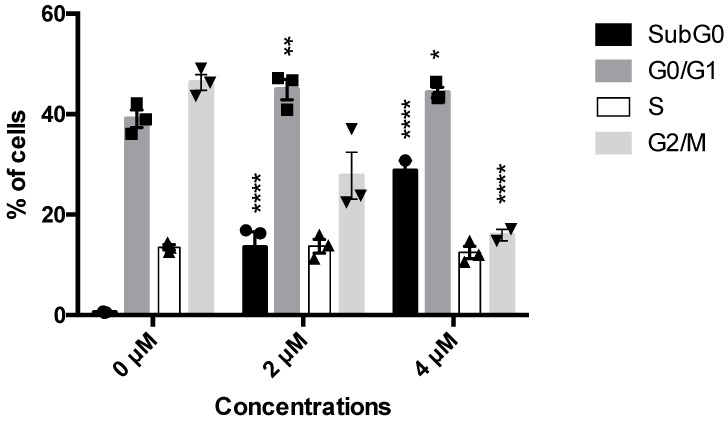
Histograms of cell-cycle distribution after Jurkat treatment with compound **5b** for 24 h. * *p* < 0.05; ** *p* < 0.01; **** *p* < 0.0001 versus untreated cells. Results are expressed as mean ± SEM of at least three independent experiments. Two-way ANOVA followed by Dunnett’s post-test was used to assess statistic and multiple comparisons.

The NCI 60 screening outlined **5b** as the most potent compound. For this reason, its antitumor and, in particular, its antileukemic potential have been investigated. Given the very favorable GI_50_, the antiproliferative effect of **5b** has been firstly explored. On Jurkat cells, at all tested concentrations, **5b** promoted a significant decrease in the cells in the G2-M phase (27.7% at 2 µM and 16.05 at 4 µM versus 47.1% of untreated cells) (Figure 1). The decrease in the number of this cell’s population was balanced by a slight increase in the percentage of cells in the G0–G1 (44.9% at 2 µM and 44.3% at 4 µM versus 37.3% of untreated cells) and by a substantial increase in cells in the subG0 phase (Figure 1), which represents cells characterized by fragmented DNA, *i.e.*, dead cells. For example, untreated cells had less than 1% of subG0 cells compared to 13.5% and 28.7%, respectively, for **5b** 2 and 4 µM (Figure 1). These interesting data prompt the following investigation about the cytotoxic potential of **5b**.

#### 2.2.4. Cytotoxic Effect on Jurkat Cells

**Figure 2:**
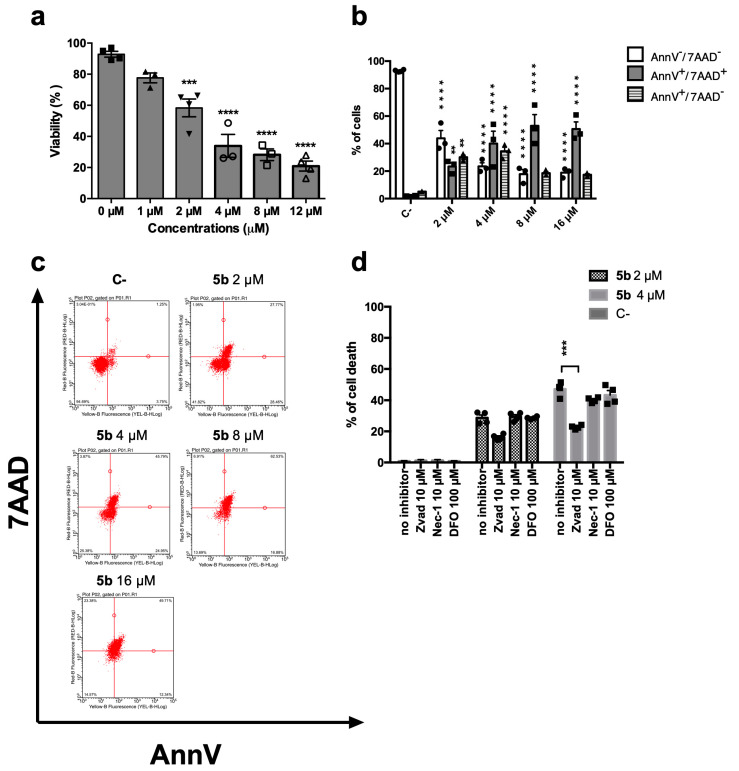
Cytotoxic effect of **5b** on Jurkat cells. (**a**) Percentage of viable cells after 24 h treatment with **5b**; (**b**) Distribution of cells treated with **5b** for 24 h in the different stages of cell death: AnnV^-^/7AAD^-^ (viable); AnnV^+^/7AAD^+^ (late stage or primary necrotic); AnnV^+^/7AAD^-^ (early phase of regulated cell death); (**c**) representative dot plots of AnnV/7AAD assay of cells treated with **5b** for 24 h; (**d**) Percentage of cell death after treatment for 24 h with **5b** with or without inhibitors of apoptosis (Zvad), necroptosis (nec-1s), or ferroptosis (DFO). ** *p* < 0.01; *** *p* < 0.001; **** *p* < 0.0001 versus untreated cells. All results are expressed as mean ± SEM of at least three independent experiments. Differences between treatments were assessed by one-way ANOVA followed by Dunnett’s post-test (**a**,**d**), or two-way ANOVA followed by Dunnett’s post-test (**b**).

**5b** induced a dose-dependent decrease in cell viability (Figure 2a). After 24 h, the IC_50_ was 3.20 µM. At the same time point, we assessed the ability of **5b** to promote regulated cell death using the exposure of phosphatidylserine (PS) as a marker (Figure 2b,c). Indeed, during the early phases of the most characterized regulated cell deaths, dying cells experience a loss in plasma membrane symmetry and translocate intracellular PS on the outer membrane as a signal to be recognized and phagocytized by antigen-presenting cells [15,16]. However, Figure 2b shows that after 24 h, **5b**-treated cells are not in the early phases of any regulated cell death, since cells experience the cell membrane rupture, as shown by the double-positive population Annexin V^+^/7-amino-actinomycin D^+^ (AnnV^+^/7AAD^+^). AnnV is the cognate ligand of PS, and 7AAD has been used as a marker of cellular membrane integrity (Figure 2b,c). This means that the double positive cells are primary or secondary necrotic, *i.e.*, in the late phases of cell death. To unravel whether the necrosis was primary or derived from an earlier cell death induction, PS exposure at an earlier time point was also investigated. After 1, 3, or 6 h, no significant PS exposure has been recorded (data not shown). PS exposure represents a universal and specific event that characterizes only regulated cell deaths, and it does not happen in necrotic cell death. Since at all time points, even just right after the treatment with the compound, cells do not experience PS exposure, but mainly membrane disruption (AnnV^+^/7AAD^+^), unregulated cell death is, at least partially, involved in the **5b** mechanism of action. To further confirm this assumption, **5b** cytotoxicity has been tested alone or together with different inhibitors of the most characterized regulated cell deaths, namely necroptosis, apoptosis, or ferroptosis. Cell death was partially restored by the pan-caspase inhibitor Zvad-fmk (Zvad), and the effect was significant only at the highest tested concentration (Figure 2d). Thus, we can conclude that **5b** induces a mixed cell death, which seems only partially regulated, in the form of apoptosis.

#### 2.2.5. Oxidative Stress and DNA Damage Analysis

**Figure 3:**
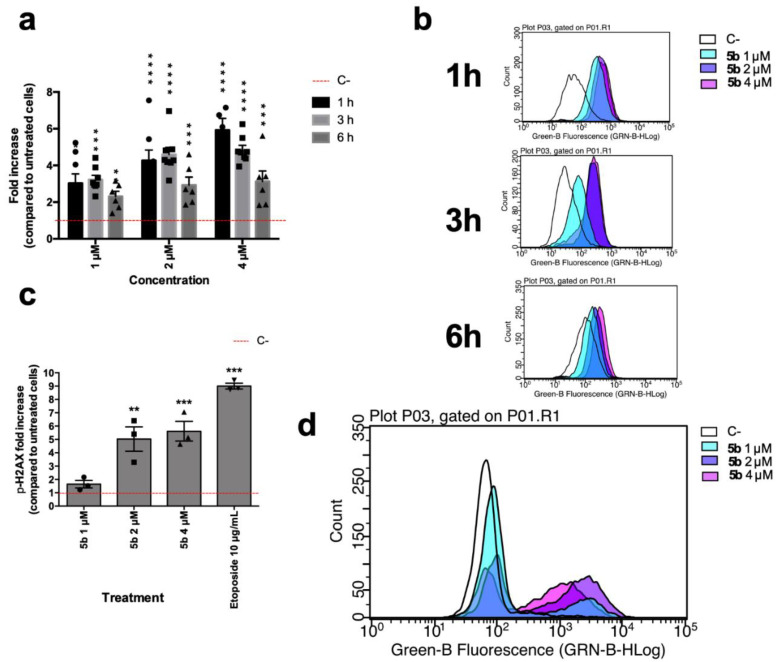
**(a**) Relative expression of intracellular levels of ROS in Jurkat cells after **5b** exposure for 1, 3, and 6 h; (**b**) Representative histograms of flow cytometric analyses of cellular ROS contents of Jurkat cells after **5b** exposure for 1, 3 and 6 h; (**c**) Relative expression of γH2Ax in Jurkat cells after 6 h of **5b** exposure. Etoposide 10 µM was used as positive control; (**d**) Representative histogram of flow cytometric analyses of γH2Ax expression of Jurkat cells after **5b** exposure. * *p* < 0.05; ** *p* < 0.01; *** *p* < 0.001; **** *p* < 0.0001 versus untreated cells. All results are expressed as mean ± SEM of at least three independent experiments. Differences between treatments were assessed by two-way ANOVA followed by Dunnett’s post-test (**a**), or one-way ANOVA followed by Dunnett’s post-test (**c**).

Brominated compounds often induce oxidative stress and can generate DNA breaks [17]; thus, to further examine the mechanism of action of **5b**, the modulation of ROS levels and ability to induce DNA damage have been investigated. Starting at a lower concentration than that of IC_50_, **5b** induced an increase in intracellular ROS levels already after 1 h (Figure 3a,b). At that time point, Jurkat cells treated with **5b** 1 µM hold around 3.03 times more ROS than untreated cells, while at the same time point, the effect of 4 µM **5b** is an increase of 5.92 compared to untreated cells (Figure 3a,b). Oxidative stress could represent a putative way through which **5b** induces the DNA damage that has been recorded. Indeed, at all tested concentrations, this compound promoted an increase in the phosphorylation of the histone H2A x (Ser139) (γH2Ax) (Figure 3c,d), which represents an early cellular response to double-strand breaks. The early DNA damage induced by **5b** could also explain why we showed that right after **5b** treatment, Jurkat cells lose membrane integrity. Indeed, early DNA damage, specifically DNA fragmentation, can lead to immediate late-stage apoptosis [18,19,20]. This type of cell death is typical of photodynamic therapy, where a photosensitizer, after specific irradiation, induces a burst of oxidative stress that generates DNA double-strand breaks that in turn kill the tumor cells [18,21]. For instance, brominated DAPI were shown to act in that way [18]. Thus, we hypothesize here that the high reactivity of **5b** could induce a similar effect without any irradiation.

## 3. Materials and Methods

### 3.1. Chemistry

The melting points are uncorrected. Elemental analyses were within ±0.4% of the theoretical values. Bakerflex plates (silica gel IB2-F) were used for TLC: the eluent was petroleum ether/acetone in various proportions. The IR spectra were recorded in nujol on a Nicolet Avatar 320 E.S.P.; ν_max_ is expressed in cm^−1^. ^1^H NMR and ^13^C-NMR spectra were recorded in (CD_3_)_2_SO on a Varian MR 400 MHz (ATB PFG probe, Crawley, United Kingdom); the chemical shift (referenced to solvent signal) is expressed in δ (ppm) and J in Hz; abbreviations: ar = aromatic, ind = indole. High-resolution mass spectrometry (HRMS) data were analyzed by flow injection, utilizing electrospray ionization (ESI) on a Waters Xevo G2-XS QTOF (Milford, MA, United States) instrument in the positive mode. Compounds were named relying on the naming algorithm developed by CambridgeSoft Corporation (Perkin Elmer, Milan, Italy) and used in Chem-BioDraw Ultra 14.0 (Perkin Elmer, Milan, Italy). ^1^H-NMR, ^13^C-NMR, and HRMS spectra are reported as Supplementary Materials. All solvents and reagents, unless otherwise stated, were supplied by Aldrich Chemical Co. Ltd. (Milan, Italy) and were used without further purification.

Indolin-2-one **1a**, 5-bromoindolin-2-one **1b** [22], 5-chloroindolin-2-one **1c**, 5-chloro-1-methylindolin-2-one **1d** [23], 5-hydroxyindolin-2-one **1e** [24], 5-hydroxy-1-methylindolin-2-one **1f** [25], 1,3-dihydro-2H-benzo[g]indol-2-one **1g** [26], 4-*tert*-butyl-2,6-diformylphenol **2**, benzene-1,3-dicarbaldehyde (isophthaldehyde) **3**, 2-chloro-1H-indole-3-carbaldehyde **6h** [27], 2-chloro-5-hydroxy-1H-indole-3-carbaldehyde **6i** [28], 2-chloro-1H-benzo[g]indole-3-carbaldehyde **6j** [9], and 1,7-dimethyl-5,7-dihydropyrrolo[3,2-f]indole-2,6(1H,3H)-dione **7** [29] are commercially available or have been prepared as described in the literature.

### 3.2. Synthesis of Compounds ***4a–g**, **5b–f**, **8h–j***

Four different methods were employed.

#### 3.2.1. Method 1 (Compounds **4a–c**, **f–g**, **5e–f**, **8h–j**)

The proper compound **1**(10 mmol) was dissolved in methanol (100 mL) and treated with the appropriate aldehyde **2** or **3** (5 mmol) and piperidine (2 mL). The reaction mixture was refluxed for 3–8 h (according to a TLC test), cooled and, if necessary, concentrated at reduced pressure or treated with water (100 mL). The yellow to orange precipitate thus formed was collected by filtration. The compounds were subjected to biological tests after crystallization from ethanol.

Since compounds **8** have an indolinone central core, the same procedure was used for their synthesis, but the stoichiometric ratios have been inverted: 5 mmol of the indolinone **7** and 10 mmol of the appropriate aldehyde **6**.

*3,3’-((5-(tert-butyl)-2-hydroxy-1,3-phenylene)bis(methaneylylidene))bis(indolin-2-one),***4a**, Yield 15%; IR (Nujol) ν_max_: 1705, 1608, 1224, 733 cm^−1^; ^1^H-NMR (DMSO–d_6_): δ 1.26 (9H, s, 3 x CH_3_), 6.82 (2H, t, ind, J = 7.6), 6.86 (2H, d, ind, J = 7.6), 7.19 (2H, t, ind, J = 7.6), 7.45 (2H, d, ind, J = 7.6), 7.71 (2H, s, ar), 7.73 (2H, s, CH), 9.88 (1H, broad, OH), 10.56 (2H, s, NH); ^13^C-NMR (DMSO-*d_6_*): 31.10, 34.28, 110.14, 120.86, 121.14, 122.41, 123.10, 127.51, 128.58, 129.99, 132.30, 142.87, 168.65; HRMS: *m/z* calculated for C_28_H_24_N_2_NaO_3_ [M + Na]^+^: 459.16846. Found: 459.16795; Anal. Calcd. for C_28_H_24_N_2_O_3_ (MW 436,51): C, 77.04; H, 5.54; N, 6.42; Found C, 77.05; H, 5.56; N, 6.39.

*3,3’-((5-(tert-butyl)-2-hydroxy-1,3-phenylene)bis(methaneylylidene))bis(5-bromoindolin-2-one),***4b**, Yield 45%; IR (Nujol) ν_max_: 1708, 1607, 1260, 1224 cm^−1^; ^1^H-NMR (DMSO-d_6_): δ 1.33 (9H, s, 3 x CH_3_), 6.85 (2H, d, ind-7, J = 8.2), 7.40 (2H, dd, ind-6, J = 8.2, J = 2.0), 7.58 (2H, d, ind-4, J = 2.0), 7.77 (2H, s, ar), 7.82 (2H, s, CH), 10.08 (1H, broad, OH), 10.77 (2H, s, NH); ^13^C-NMR (DMSO-*d_6_*): δ 31.48, 34.76, 112.41, 112.99, 123.29, 123.71, 125.05, 127.07, 129.45, 132.57, 134.59, 142.35, 168.57; HRMS: *m/z* calculated for C_28_H_23_Br_2_N_2_O_3_ [M + H]^+^: 593.00754. Found: 595.00656; Anal. Calcd for C_28_H_22_Br_2_N_2_O_3_ (MW: 594,30): C, 56.59; H, 3.73; N, 4.71; Found C, 56.57; H, 3.75; N, 4.74.

3,3’-((5-(tert-butyl)-2-hydroxy-1,3-phenylene)bis(methaneylylidene))bis(5-chloroindolin-2-one), **4c**, Yield 60%; IR (Nujol) ν_max_: 1707, 1607, 1312, 1260 cm^−1^; ^1^H-NMR (DMSO-d_6_): δ 1.32 (9H, s, 3 x CH_3_), 6.90 (2H, d, ind-7, J = 8.0), 7.28 (2H, dd, ind-6, J = 8.0, J = 2.0), 7.44 (2H, d, ind-4, J = 2.0), 7.77 (2H, s, ar), 7.83 (2H, s, CH), 10.08 (1H, broad, OH), 10.74 (2H, s, NH); ^13^C-NMR (DMSO-*d_6_*): δ 31.00, 34.33, 111.46, 121.93, 122.80, 124.83, 129.15, 134.20, 141.56, 168.29; HRMS: *m/z* calculated for C_28_H_23_Cl_2_N_2_O_3_ [M + H]^+^: 505.10857. Found: 505.10852; Anal. Calcd for C_28_H_22_Cl_2_N_2_O_3_ (MW: 505,40): C, 66.54; H, 4.39; N, 5.54; Found C, 66.57; H, 4.36; N, 5.52.

*3,3’-((5-(tert-butyl)-2-hydroxy-1,3-phenylene)bis(methaneylylidene))bis(5-hydroxy-1-methylindolin-2-one),***4f**, Yield 15%; IR (Nujol) ν_max_: 1677, 1650, 1595, 1213 cm^−1^; ^1^H-NMR (DMSO-d_6_): δ 1.30 (9H, s, 3 x CH_3_), 3.17 (6H, s, CH_3_), 6.75 (2H, dd, ind-6, J = 8.0, J = 2.4), 6.85 (2H, d, ind-7, J = 8.0), 7.04 (2H, d, ind-4, J = 2.4), 7.75 (2H, s, ar), 7.76 (2H, s, CH), 9.02 (2H, s, OH), 9.92 (1H, s, OH); ^13^C-NMR (DMSO-*d_6_*): δ 25.95, 30.98, 34.31, 108.75, 110.37, 116.16, 121.21, 122.43, 122.95, 127.24, 128.68, 132.52, 136.56, 152.47, 167.06; HRMS: *m/z* calculated for C_30_H_29_N_2_O_5_ [M + H]^+^: 497.20765. Found: 497.20733; Anal. Calcd for C_30_H_28_N_2_O_5_ (MW: 496,56): C, 72.56; H, 5.68; N, 5.64; Found C, 72.58; H, 5.65; N, 5.63.

*3,3’-((5-(tert-butyl)-2-hydroxy-1,3-phenylene)bis(methaneylylidene))bis(1,3-dihydro-2H-benzo[g]indol-2-one),***4g**, Yield 10%; IR (Nujol) ν_max_: 1701, 1614, 1311, 1265 cm^−1^; ^1^H-NMR (DMSO-d_6_): δ 1.33 (9H, s, 3 x CH_3_), 7.44 (2H, d, ind, J = 8.4), 7.53 (4H, m, ind), 7.64 (2H, d, ind, J = 8.4), 7.85 (2H, s, ar), 7.86 (2H, s, CH), 7.88 (2H, m, ind), 8.15 (2H, m, ind), 10.04 (1H, broad, OH), 10.41 (2H, s, NH); ^13^C-NMR (DMSO-*d_6_*): δ 30.52, 34.25, 114.89, 119.41, 120.31, 122.61, 123.26, 127.01, 128.18, 128.45, 128.89, 132.07, 133.91, 139.99, 169.73; HRMS: *m/z* calculated for C_36_H_28_N_2_NaO_3_ [M + Na]^+^: 559.19976. Found: 559.19979; Anal. Calcd for C_36_H_28_N_2_O_3_ (MW: 536,63): C, 80.58; H, 5.26; N, 5.22; Found C, 80.59; H, 5.28; N, 5.19.

*3,3’-(1,3-phenylenebis(methaneylylidene))bis(5-hydroxyindolin-2-one),***5e**, Yield 20%; IR (Nujol) ν_max_: 1701, 1614, 1311, 1265 cm^−1^; ^1^H-NMR (DMSO-d_6_): δ 6.67 (4H, m, ind-6+ind-7), 7.02 (2H, d, ind-4, J = 1.6), 7.62 (2H, s, CH), 7.68 (1H, t, ar, J = 8), 7.79 (1H, d, ar, J = 8), 7.88 (1H, s, ar), 9.04 (2H, s, OH), 10.31 (2H, s, NH); ^13^C-NMR (DMSO-*d_6_*): δ 109.97, 110.63, 117.01, 121.40, 129.02, 129.17, 129.89, 134.61, 13.07, 135.46, 151.87, 168.57; HRMS: *m/z* calculated for C_24_H_17_N_2_O_4_ [M + H]^+^: 397.11883. Found: 397.11863; Anal. Calcd for C_24_H_16_N_2_O_4_ (MW: 396,40): C, 72.72; H, 4.07; N, 7.07; Found C, 72.70; H, 4.05; N, 7.09.

*3,3’-(1,3-phenylenebis(methaneylylidene))bis(5-hydroxy-1-methylindolin-2-one),***5f**, Yield 30%; IR (Nujol) ν_max_: 1678, 1598, 1217, 1118 cm^−1^; ^1^H-NMR (DMSO-d_6_): δ 3.15 (6H, s, CH_3_), 6.74 (2H, dd, ind-6, J = 8.0, J = 2.0), 6.85 (2H, d, ind-7, J = 8.0), 7.07 (2H, d, ind-4, J = 2.0), 7.69 (1H, t, ar, J = 7.4), 7.72 (2H, s, CH), 7.81 (2H, d, ar, J = 7.4), 7.91 (1H, s, ar), 9.15 (2H, s, OH); HRMS: *m/z* calculated for C_26_H_21_N_2_O_4_ [M + H]^+^: 425.15013. Found: 425.15010; Anal. Calcd for C_26_H_20_N_2_O_4_ (MW: 424,46): C, 73.57; H, 4.75; N, 6.60; Found C, 73.55; H, 4.76; N, 6.61

*3,5-bis((2-chloro-1H-indol-3-yl)methylene)-1,7-dimethyl-5,7-dihydropyrrolo[3,2-f]indole-2,6(1H,3H)-dione,***8h**, Yield 20%; IR (Nujol) ν_max_: 1681, 1604, 1271, 1127 cm^−1^; ^1^H-NMR (DMSO-d_6_): δ 3.30 (6H, s, CH_3_), 6.38 (1H, s, ar), 6.45 (2H, d, ind, J=7.6), 6.59 (2H, t, ind, J = 7.6), 6.87 (2H, t, ind, J = 7.6), 6.91 (1H, s, ar), 7.15 (2H, d, ind, J = 7.6), 7.37 (2H, s, CH), 12.16 (2H, s, NH); ^13^C-NMR (DMSO-*d_6_*): δ 26.26, 90.52, 106.53, 11.50, 113.74, 119.18, 120.10, 121.50, 122.88, 123.12, 123.98, 126.18, 134.23, 144.53, 168.09, HRMS: *m/z* calculated for C_30_H_21_Cl_2_N_4_O_2_ [M + H]^+^: 539.10416. Found: 539.10237; Anal. Calcd for C_30_H_20_Cl_2_N_4_O_2_ (MW: 539,42): C, 66.80; H, 3.74; N, 10.39; Found C, 66.83; H, 3.71; N, 10.38.

*3,5-bis((2-chloro-5-hydroxy-1H-indol-3-yl)methylene)-1,7-dimethyl-5,7-dihydropyrrolo[3,2-f]indole-2,6(1H,3H)-dione,***8i**, Yield 15%; IR (Nujol) ν_max_: 1678, 1609, 1273, 1134, 1108 cm^−1^; ^1^H-NMR (DMSO-d_6_): δ 3.32 (6H, s, CH_3_), 5.82 (2H, d, ind-4, J = 1.9), 6.40 (2H, dd, ind-6, J = 8.8, J = 1.9), 6.47 (1H, s, ar), 6.89 (1H, s, ar), 6.93 (2H, d, ind-7, J = 8.8), 7.38 (2H, s, CH), 8.42 (2H, s, OH), 11.87 (2H, s, NH); ^13^C-NMR (DMSO-*d_6_*): δ 26.25, 90.20, 104.50, 106.09, 11.39, 112.05, 114.09, 123.17, 123.66, 124.20, 125.85, 128.39, 144.02, 151.41, 168.28; HRMS: *m/z* calculated for C_30_H_21_Cl_2_N_4_O_4_ [M + H]^+^: 571.09399. Found: 571.09044; Anal. Calcd for C_30_H_20_Cl_2_N_4_O_4_ (MW: 571,41): C, 63.06; H, 3.53; N, 9.81; Found C, 63.08; H, 3.50; N, 9.82.

*3,5-bis((2-chloro-1H-benzo[g]indol-3-yl)methylene)-1,7-dimethyl-5,7-dihydropyrrolo[3,2-f]indole-2,6(1H,3H)-dione,***8j**, Yield 15%; IR (Nujol) ν_max_: 1659, 1603, 1269, 1125cm^−1^; ^1^H-NMR (DMSO-d_6_): δ 3.33 (6H, s, CH_3_), 6.69 (2H, d, ind, J = 8), 6.89 (1H, s, ar,), 6.94 (1H, s, ar,), 7.03 (2H, d, ind, J = 8), 7.35 (2H, t, ind, J = 8), 7.43 (2H, s, CH), 7.47 (2H, t, ind, J = 8), 7.61 (2H, d, ind, J = 8), 7.78 (2H, d, ind, J = 8), 12.03 (2H, s, NH); ^13^C-NMR (DMSO-*d_6_*): δ 26.33, 108.18, 113.72, 119.44, 119.60, 119.96, 120.34, 120.82, 123.01, 123.39, 123.69, 124.12, 125.48, 127.60, 129.18, 129.21, 144.76, 168.21; HRMS: *m/z* calculated for C_38_H_25_Cl_2_N_4_O_2_ [M + H]^+^: 639.13546. Found: 639.13362; Anal. Calcd for C_38_H_24_Cl_2_N_4_O_2_ (MW: 639,54): C, 71.37; H, 3.78; N, 8.76; Found C, 71.33; H, 3.80; N, 8.78

#### 3.2.2. Method 2 (Compounds **4d–e**)

The appropriate oxindole **1** (10 mmol) was dissolved in ethanol (100 mL) and treated with the aldehyde **2** (5 mmol) and 37% hydrochloric acid (1 mL). The reaction mixture was refluxed for 2-4 h (according to a TLC test) and cooled. The yellow to orange precipitates thus formed were collected by filtration. The crude products were purified by crystallization with ethanol (**4d**) or were treated with diethyl ether (**4e**) to give the desired products.

*3,3’-((5-(tert-butyl)-2-hydroxy-1,3-phenylene)bis(methaneylylidene))bis(5-chloro-1-methylindolin-2-one),***4d**, Yield 25%; IR (Nujol) ν_max_: 1706, 1603, 1268, 1108 cm^−1^; ^1^H-NMR (DMSO-d_6_): δ 3.21 (6H, s, CH_3_), 7.11 (2H, d, ind-7, J = 8.0), 7.31 (2H, d, ind-4, J = 2.0), 7.38 (2H, dd, ind-6, J = 8.0, J = 2.0), 7.75 (1H, t, ar, J = 8.4), 7.83 (2H, d, ar, J = 8.4), 7.85 (2H, s, CH), 7.92 (1H, s, ar); ^13^C-NMR (DMSO-*d_6_*): δ 26.11, 30.92, 34.33, 110.34, 121.69, 122.03, 122.77, 125.52, 125.85, 129.19. 129.28, 134.69, 142.72, 166.81; HRMS: *m/z* calculated for C_30_H_27_Cl_2_N_2_O_3_ [M + H]^+^: 533.13987. Found: 533.13988; Anal. Calcd for C_30_H_26_Cl_2_N_2_O_3_ (MW: 533,45): C, 67.55; H, 4.91; N, 5.25; Found C, 67.56; H, 4.93; N, 5.22

*3,3’-((5-(tert-butyl)-2-hydroxy-1,3-phenylene)bis(methaneylylidene))bis(6-hydroxyindolin-2-one),***4e**, Yield 15%; IR (Nujol) ν_max_: 1693, 1614, 1259, 1198 cm^−1^; ^1^H-NMR (DMSO-d_6_): δ 1.31 (9H, s, 3 x CH_3_), 6.66 (4H, m, ind-4 + ind-6), 7.00 (2H, s, ar), 7.69 (2H, s, ind-7), 7.74 (2H, s, CH), 8.91 (2H, s, OH), 9.82 (1H, s, OH), 10.27(2H, s, NH); HRMS: *m/z* calculated for C_28_H_24_N_2_NaO_5_ [M + Na]^+^: 491.15829. Found: 491.15760; Anal. Calcd for C_28_H_24_N_2_O_5_ (MW: 468,51): C, 71.78; H, 5.16; N, 5.98; Found C, 71.76; H, 5.17; N, 5.99.

#### 3.2.3. Method 3 (Compound **5b**)

The 5-bromoindolin-2-one **1b** (10 mmol) was dissolved in acetic acid (50 mL) and treated with isophthaldehyde **3** (5 mmol) and 37% hydrochloric acid (1 mL). The reaction mixture was refluxed for 4 h and the solid separated on cooling was collected by filtration. The crude product was purified by crystallization with ethanol.

*3,3’-(1,3-phenylenebis(methaneylylidene))bis(5-bromoindolin-2-one),***5b**, Yield 10%; IR (Nujol) ν_max_: 1712, 1611, 1306, 810 cm^−1^; ^1^H-NMR (DMSO-d_6_): δ 6.81 (2H, d, ind-7, J = 8.4), 7.36 (2H, dd, ind-6, J = 8.4, J = 1.9), 7.39 (2H, d, ind-4, J = 1.9), 7.70 (3H, m, 1ar + CH), 7.78 (2H, d, ar, J = 6.8), 7.88 (1H, s, ar), 10.76 (2H, s, NH); HRMS: *m/z* calculated for C_24_H_15_Br_2_N_2_O_2_ [M + H]^+^: 520.95003. Found: 522.94752; Anal. Calcd for C_24_H_14_Br_2_N_2_O_2_ (MW: 522,20): C, 55.20; H, 2.70; N, 5.36; Found C, 55.18; H, 2.71; N, 5.35.

#### 3.2.4. Method 4 (Compounds **5c–d**)

Isophthaldehyde **3** (5 mmol) was dissolved in toluene (75 mL) and treated with the appropriate oxindole **1** (10 mmol) in the presence of 4-toluenesulfonic acid (0.5 mmol). The reaction mixture was refluxed for 1 h, and after cooling, the precipitate formed was collected by filtration. The crude derivatives were crystallized from ethanol (**5c**) or toluene (**5d**).

*3,3’-(1,3-phenylenebis(methaneylylidene))bis(5-chloroindolin-2-one),***5c**, Yield 30%; IR (Nujol) ν_max_: 1735, 1713, 1690, 800 cm^−1^; ^1^H-NMR (DMSO-d_6_): δ 6.85 (2H, d, ind-7, J = 8), 7.23 (2H, m, ind-4), 7.25(2H, m, ind-6), 7.71 (1H, m, ar), 7.73 (2H, s, CH), 7.79 (2H, d, ar, J = 8), 7.88 (1H, s, ar), 10.75 (2H, s, NH); HRMS: *m/z* calculated for C_24_H_14_Cl_2_N_2_NaO_2_ [M + Na]^+^: 455.03300. Found: 455.03226; Anal. Calcd for C_24_H_14_Cl_2_N_2_O_2_ (MW: 433,29): C, 66.53; H, 3.26; N, 6.47; Found C, 66.55; H, 3.27; N, 6.43.

*3,3’-(1,3-phenylenebis(methaneylylidene))bis(5-chloro-1-methylindolin-2-one),***5d**, Yield 60%; IR (Nujol) ν_max_: 1725, 1607, 1109, 796 cm^−1^; ^1^H-NMR (DMSO-d_6_): δ 3.21 (6H, s, CH_3_), 7.09(2H, d, ind-7, J = 8.0), 7.31 (2H, d, ind-4, J = 2.0), 7.38 (2H, dd, ind-6, J = 8.0, J = 2.0), 7.75 (1H, t, ar, J = 8.4), 7.83 (2H, d, ar, J = 8.4), 7.85 (2H, s, CH), 7.92 (1H, s, ar); HRMS: *m/z* calculated for C_26_H_19_Cl_2_N_2_O_2_ [M + H]^+^: 461.08236. Found: 461.08213; Anal. Calcd for C_26_H_18_Cl_2_N_2_O_2_ (MW: 461,34): C, 67.69; H, 3.93; N, 6.07; Found C, 67.67; H, 3.95; N, 6.08.

### 3.3. NCI Screening

To test the cytostatic and cytotoxic impact of the synthetic compounds, the NCI-60 Human Tumor Cell Lines Screening has been used. Leukemia, Melanoma, NSCLC, Colon, CNS, Ovarian, Renal, and Breast cancer cells (https://dtp.cancer.gov/discovery_development/nci-60/cell_list.htm, accessed on 15 August 2021) were seeded in a 96 multi-well plate at a cell density of 5000–40000 cells/well, depending on the cell line, as described [30]. Compounds subjected to the full 5-concentration assay (**4a**, **4b**, **4c**, **4d**, **4f**, **4g**, **5b**, **5c**, **5d**, **5e**, **5f**, **8h**) and vincristine sulfate were diluted in fresh media and added to the cells at scalar concentration 0–1000 µM or 0–100 µM. Briefly, endpoint determinations of the cell viability or cell growth were performed at 48 h of treatment by in situ fixation of cells, which was followed by staining with a protein-binding dye, sulforhodamine B (SRB) (https://dtp.cancer.gov/discovery_development/nci-60/methodology.htm accessed on 15 August 2021). After washing, SRB optical density was measured spectrophotometrically. TGI, LC_50_, and GI_50_ were established according to the NCI screening procedures (https://dtp.cancer.gov/discovery_development/nci-60/methodology.htm accessed on 15 August 2021).

### 3.4. Cell Culture

Jurkat cells were purchased from ATCC and cultured in RPMI 1640 supplemented with 10% heat-inactivated fetal calf serum, 1% antibiotics (penicillin 5000 IU/streptomycin 5 mg/mL), and 1% L-glutamine solution (all purchased from Biochrome, Cambridge, UK). Cultured cells were maintained in 5% CO_2_ and humidified air at 37 °C.

### 3.5. Analysis of Cell Cycle

After treatment with **5b** for 24 h, which represents the doubling time of Jurkat cells, Jurkat were fixed with 70% ice-cold ethanol and, after washing, suspended in 200 μL of Guava cell cycle reagent (Merck Millipore, Burlington, MA, USA), containing propidium iodide. At the end of incubation at room temperature for 30 min in the dark, samples were analyzed via flow cytometry.

### 3.6. Analysis of Cell Viability

Jurkat cells were treated with increasing concentrations of **5b** for 24 h and analyzed with the Guava ViaCount Reagent (Merck Millipore, Burlington, MA, USA), following the manufacturer’s instructions. Briefly, cells were diluted with the reagent containing 7-AAD and incubated at room temperature in the dark for 5 min before been recorded at the flow cytometer. The IC_50_ (the half-maximal inhibitory concentration) was calculated by interpolation from the nonlinear dose–response curve.

Cell death analysis with or without specific inhibitors on Jurkat cells was performed as follows. Briefly, cells were pretreated for 1 h with or without the pan-caspase inhibitor carbobenzoxy-valyl-alanyl-aspartyl-[O-methyl]-fluoromethylketone (zVAD-fmk, 10 μM, Bachem, Bubendorf, Switzerland), the RIPK1 inhibitor necrostatin-1 s (Nec1s, 10 μM, Abcam), the iron chelator deferoxamine (DFO, 10 μM, Sigma-Aldrich, St. Louis, MO, USA) to respectively block apoptosis, necroptosis, or ferroptosis. Then, Jurkat cells were treated for 24 h with compound **5b**, and cell death was analyzed through the fluorescent apoptosis/necrosis (FAN) assay [31] using the cell-impermeant fluorescent nuclear probe Sytox Green (Thermo Fisher Scientific, Waltham, MA, USA). Fluorescence was measured on a Tecan infinite 200 microplate reader.

### 3.7. Annexin V assay

After 1, 3, 6, or 24 h treatments with **5b**, aliquots of 2 × 10^4^ Jurkat were resuspended in 100 μL of RPMI 1640 containing at least 10% of FBS and stained with an equal volume of Guava Nexin Reagent (Merck KGaA, Darmstadt, Germany) containing annexin V-phycoerythrin and 7-amino-actinomycin D (7-AAD) and analyzed via flow cytometry.

### 3.8. Detection of Intracellular ROS

1, 3, or 6 h after **5b** treatment, cells were incubated with 2′,7′-dichlorodihydro- fluorescein diacetate (H2DCFDA, 1 μM) (Sigma-Aldrich, St. Louis, MO, USA) for 20 min at 37 °C, 5% CO_2_ in the dark. Cells were washed twice and analyzed via flow cytometry.

### 3.9. DNA Damage Analysis

Phosphorylation of histone γH2Ax was used as a marker of **5b** genotoxic potential. After 6 h of treatment with **5b**, cells were fixed, permeabilized, and incubated for 30 min in the dark at room temperature with an anti-γ-H2AX (Ser139) antibody clone JBW301, FITC conjugate (Merck Millipore 16-202A, Burlington, MA, USA). Etoposide 10 μM was used as a positive control. Samples were analyzed via flow cytometry.

### 3.10. Flow Cytometry

EasyCyte 5HT (Merck Millipore, Burlington, MA, USA) was used to perform all flow cytometric analyses. For each sample, at least 10,000 events were recorded.

### 3.11. Statistical Analysis

All results are expressed as mean ± SEM of at least three independent experiments. Differences between treatments were assessed by t-test, one-way ANOVA, or two-way ANOVA, followed by Dunnett’s post-test. All statistical analyses were performed using GraphPad InStat 6.0 version (GraphPad Prism, San Diego, CA, USA). *p* < 0.05 was considered significant.

## 4. Conclusions

This study reports the design, synthesis, and in vitro antitumor evaluation of a panel of new bis-indolinone derivatives. After the primary screening, compound **5b** proved to be the most potent of the series and was chosen to investigate its antileukemic potential. In addition, **5b** was shown to induce a mixed type of cell death, which was apparently only partially regulated. Further studies will be needed to confirm that **5b** causes a mix of apoptosis and direct late stages of this regulated cell-death type and to directly correlate the increased ROS levels with DNA damage and cell death. Altogether, the data obtained in the study depict a promising antileukemic profile of **5b** and corroborate the rationale of the synthesis of this library of compounds. In addition, since the chemical structure seems suitable, in the future, it could be interesting to apply photodynamic therapy on **5b**, using it as a photosensitizer, trusting for an increase in potency.

## Figures and Tables

**Table 1:** Nine subpanels at five concentrations: growth inhibition, cytostatic and cytotoxic activity (μM) of the selected compounds.

Comp ^a^	Modes	Leukemia	NSCLC	Colon	CNS	Melanoma	Ovarian	Renal	Prostate	Breast	MG-MID ^b^
**4a**	**GI_50_**	3.98	6.76	4.68	4.37	4.90	6.76	4.90	5.01	5.62	5.13
	**TGI**	21.38	20.89	16.60	17.38	16.60	20.42	18.62	16.60	18.20	18.62
	**LC_50_**	79.43	51.29	43.65	45.71	45.71	53.70	47.86	43.65	50.12	51.29
**4b** ^c^	**GI_50_**	2.69	3.98	4.27	2.19	2.40	2.75	2.40	3.09	2.45	2.88
	**TGI**	11.48	13.80	13.80	7.94	7.08	11.48	11.48	13.18	7.76	10.47
	**LC_50_**	75.86	41.69	38.90	63.10	20.42	44.67	33.11	43.65	30.20	36.31
**4c**	**GI_50_**	1.86	2.57	2.45	1.29	1.66	1.82	2.24	1.95	1.82	1.95
	**TGI**	7.59	7.59	6.76	3.31	3.89	5.13	7.59	4.79	4.79	5.62
	**LC_50_**	51.29	20.89	21.88	8.32	9.33	20.89	22.39	15.49	17.38	18.20
**4d**	**GI_50_**	2.04	2.51	2.57	1.78	1.82	2.40	3.24	3.31	2.40	2.63
	**TGI**	12.59	8.13	8.71	8.32	4.27	8.91	10.23	13.18	12.02	8.51
	**LC_50_**	93.33	28.84	28.18	38.90	12.02	30.90	32.36	45.71	46.77	30.90
**4f** ^c^	**GI_50_**	1.48	3.55	2.95	2.88	2.45	4.27	4.37	2.14	3.31	3.09
	**TGI**	8.91	10.72	8.13	9.77	6.92	15.49	12.88	9.12	15.14	10.47
	**LC_50_**	100.00	33.88	22.91	33.88	23.44	46.77	31.62	37.15	51.29	37.15
**4g**	**GI_50_**	3.31	7.94	11.22	6.76	7.41	7.94	12.30	6.76	5.75	7.76
	**TGI**	20.42	30.90	45.71	38.90	27.54	50.12	51.29	70.79	50.12	38.90
	**LC_50_**	97.72	79.43	79.43	83.18	79.43	79.43	85.11	100.00	95.50	83.18
**5b** ^c^	**GI_50_**	0.51	1.62	0.63	1.10	0.72	1.35	0.98	1.10	0.74	0.91
	**TGI**	3.16	8.32	1.95	4.57	2.29	6.61	3.63	5.25	5.89	4.07
	**LC_50_**	61.66	43.65	7.59	19.50	8.91	31.62	16.22	25.70	23.99	21.38
**5c**	**GI_50_**	0.56	2.04	0.89	1.05	0.89	1.62	1.23	2.04	0.89	1.15
	**TGI**	5.50	7.59	2.19	3.98	2.19	7.08	3.72	12.88	4.68	4.27
	**LC_50_**	100.00	25.12	5.01	17.38	5.01	19.50	12.88	50.12	12.30	14.79
**5d** ^c^	**GI_50_**	0.55	2.95	1.58	2.19	2.09	2.95	2.75	2.51	2.00	2.04
	**TGI**	6.61	29.51	5.01	12.88	6.76	13.18	22.91	45.71	12.02	12.88
	**LC_50_**	100.00	77.62	20.89	56.23	25.70	75.86	89.13	100.00	66.07	57.54
**5e**	**GI_50_**	2.95	3.98	2.63	2.19	2.14	4.79	6.17	2.69	2.57	3.24
	**TGI**	33.11	16.98	10.00	6.46	4.79	15.49	30.20	9.77	7.24	12.30
	**LC_50_**	100.00	47.86	44.67	28.18	15.85	54.95	69.18	56.23	41.69	45.71
**5f**	**GI_50_**	0.87	2.69	1.29	1.66	1.74	2.45	2.40	1.62	1.74	1.82
	**TGI**	11.22	7.08	2.88	3.72	3.55	7.24	4.90	3.39	5.13	5.01
	**LC_50_**	100.00	21.88	8.13	8.71	7.94	23.99	10.47	7.08	21.88	15.14
**8h** ^c^	**GI_50_**	5.49	5.54	5.54	5.59	5.39	5.30	5.55	5.30	5.47	5.48
	**TGI**	4.46	4.26	4.44	4.37	4.29	4.12	4.28	4.16	4.21	4.29
Vincristinesulfate ^d^	**GI_50_**	0.10	0.25	0.10	0.13	0.16	0.32	0.32	0.13	0.32	0.20
	**TGI**	15.85	15.85	3.98	6.31	7.94	19.95	19.95	6.31	7.94	10.00

^a^ Highest conc. = 100 μM: only modes showing a value < 100 μM are reported. The compound exposure time was 48 h, ^b^ Mean graph midpoint, i.e., the mean concentration for all cell lines, ^c^ Mean of two separate experiments, ^d^ Highest conc. = 10^−3^M.

## Data Availability

The data presented in this study are available in this article.

## Supplementary Materials

The following are available online, ^1^H NMR and ^13^C NMR spectra; HRMS spectra.

## Sample Availability

Samples of the compounds are available from the corresponding author.
