# Supplementary material for: Synthesis and Biological Evaluation of New Bis-Indolinone Derivatives Endowed with Cytotoxic Activity
Source: Molecules. 2021 Oct 16;26(20):6277. doi: 10.3390/molecules26206277 (PMC8537952; doi:10.3390/molecules26206277)
Supplement: Supplementary file 1 [file molecules-26-06277-s001.zip › molecules-1402803-supplementary.pdf]

molecules

Synthesis and biological evaluation of new bis-indolinone derivatives endowed with cytotoxic activity

Rita Morigi [1,†], Elena Catanzaro [2,†], Alessandra Locatelli [1,*], Cinzia Calcabrini [2], Valentina Pellicioni [2], Alberto Leoni [1] and Carmela Fimognari [2]

[1] Department of Pharmacy and Biotechnology, Alma Mater Studiorum-University of Bologna, Via Belmeloro 6, 40126 Bologna, Italy; rita.morigi@unibo.it (R.M.); alberto.leoni@unibo.it (A.L.)
[2] Department for Life Quality Studies, Alma Mater Studiorum–University of Bologna, Corso d'Augusto 237, 47921 Rimini, Italy; elena.catanzaro2@unibo.it (E.C.); ccalcabrini@hotmail.com (C.C.); valentina.pellicion2@unibo.it (V.P.); carmela.fimognari@unibo.it (C.F.)
* Correspondence: alessandra.locatelli@unibo.it; Tel.: +39-0512099712
† These authors contributed equally to this work

Contents:

^{1}H NMR and ^{13}C NMR spectra……………………..……………………………………....pag. 2-15

HRMS spectra…………………………………………………………………………...pag. 16-20

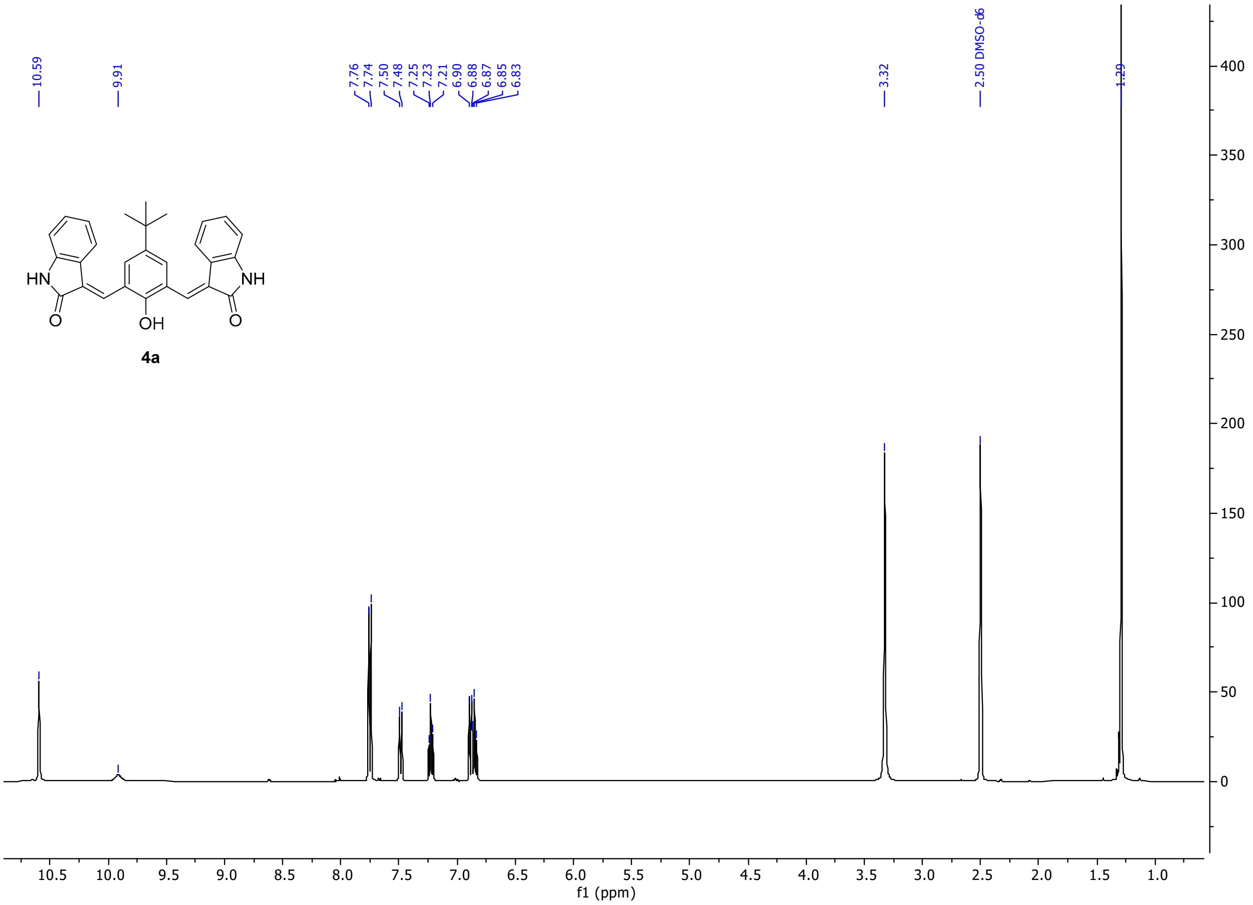
10.59
9.91
7.76
7.74
7.50
7.48
7.25
7.23
7.21
6.90
6.88
6.87
6.85
6.83
3.32
2.50 DMSO-d6
1.29
HN
NH
OH
O
4a
f1 (ppm)

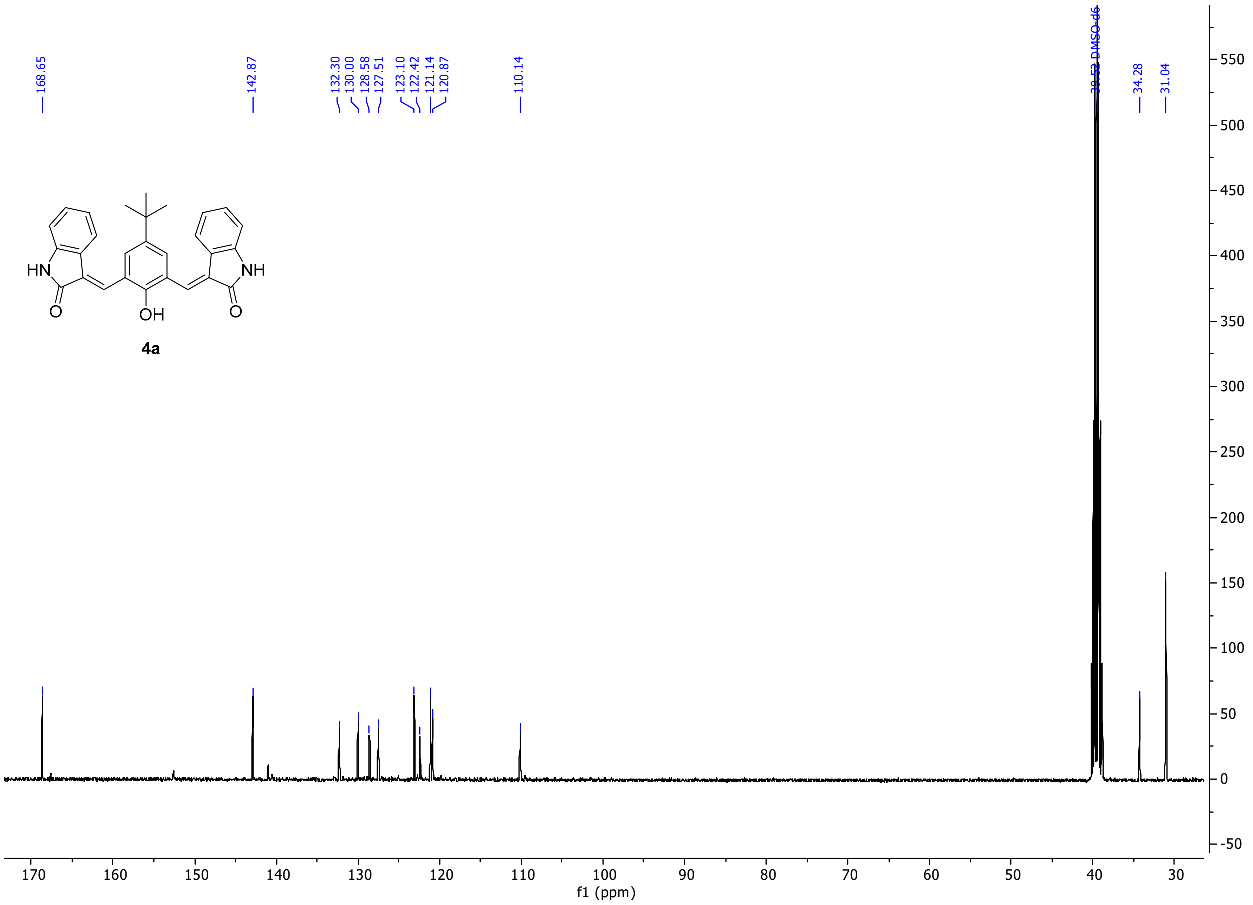
168.65
142.87
132.30
130.00
128.58
127.51
123.10
122.42
121.14
120.87
110.14
39.52 DMSO-d6
34.28
31.04
HN
NH
OH
O
4a
f1 (ppm)

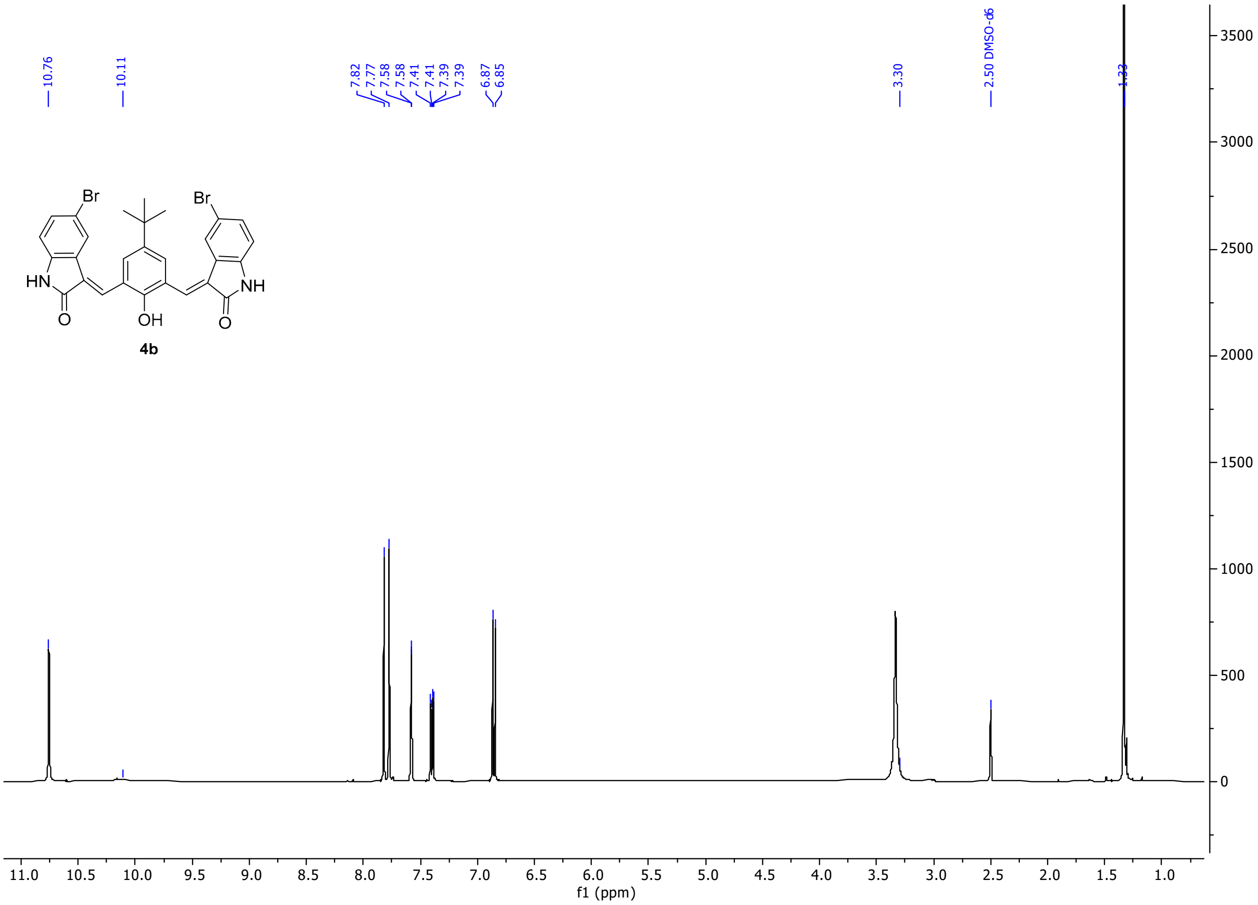
10.76
10.11
7.82
7.77
7.58
7.58
7.41
7.41
7.39
7.39
6.87
6.85
3.30
2.50 DMSO-d6
1.33
Br
Br
HN
NH
O
OH
O
4b
3500
3000
2500
2000
1500
1000
500
0
11.0 10.5 10.0 9.5 9.0 8.5 8.0 7.5 7.0 6.5 6.0 5.5 5.0 4.5 4.0 3.5 3.0 2.5 2.0 1.5 1.0
f1 (ppm)

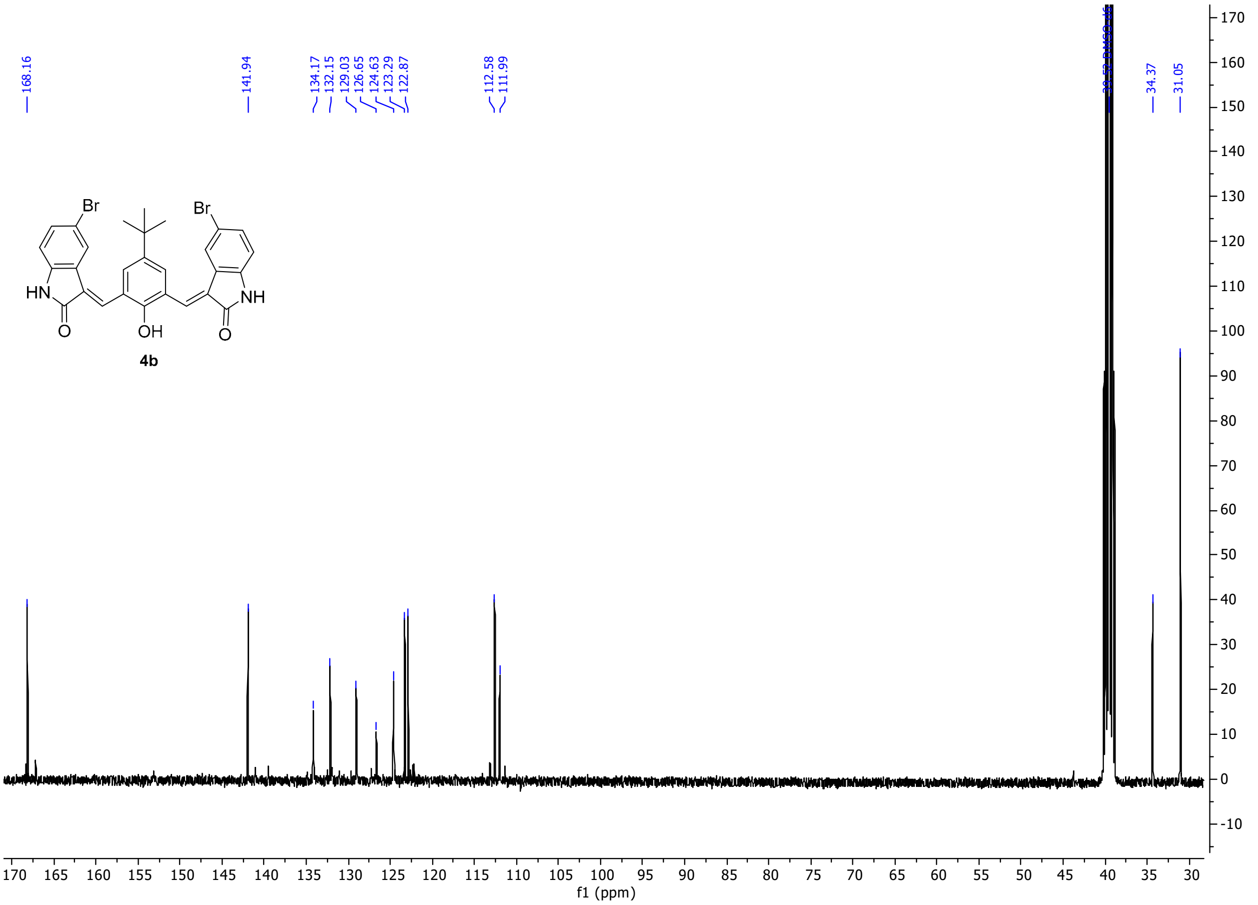
168.16
141.94
134.17
132.15
129.03
126.65
124.63
123.29
122.87
112.58
111.99
34.37
31.05
Br
Br
HN
NH
O
OH
O
4b
170
160
150
140
130
120
110
100
90
80
70
60
50
40
30
20
10
0
-10
170 165 160 155 150 145 140 135 130 125 120 115 110 105 100 95 90 85 80 75 70 65 60 55 50 45 40 35 30
f1 (ppm)

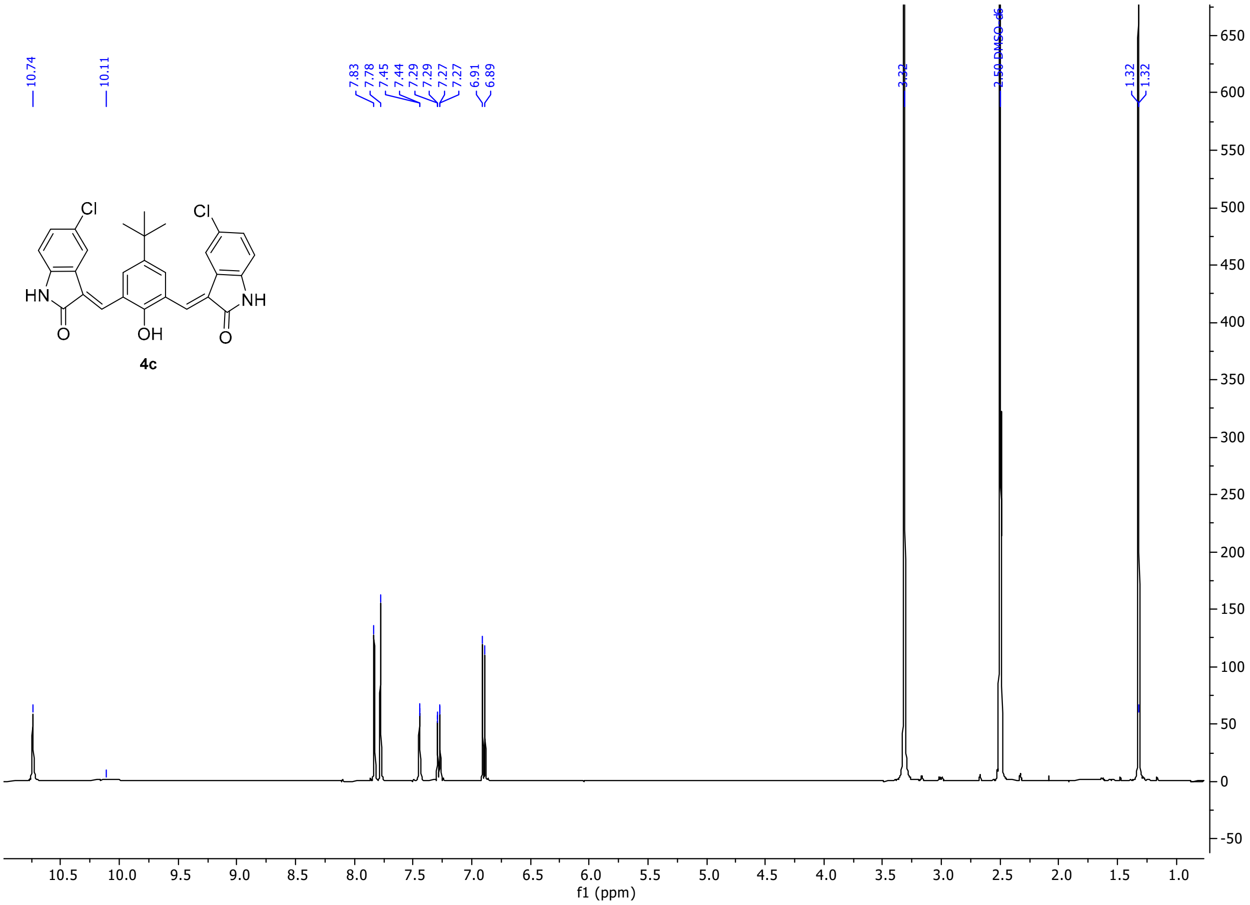
10.74
10.11
7.83
7.78
7.45
7.44
7.29
7.29
7.27
7.27
6.91
6.89
3.32
2.50 DMSO-d6
1.32
1.32
Cl
Cl
HN
NH
O
OH
O
4c
f1 (ppm)

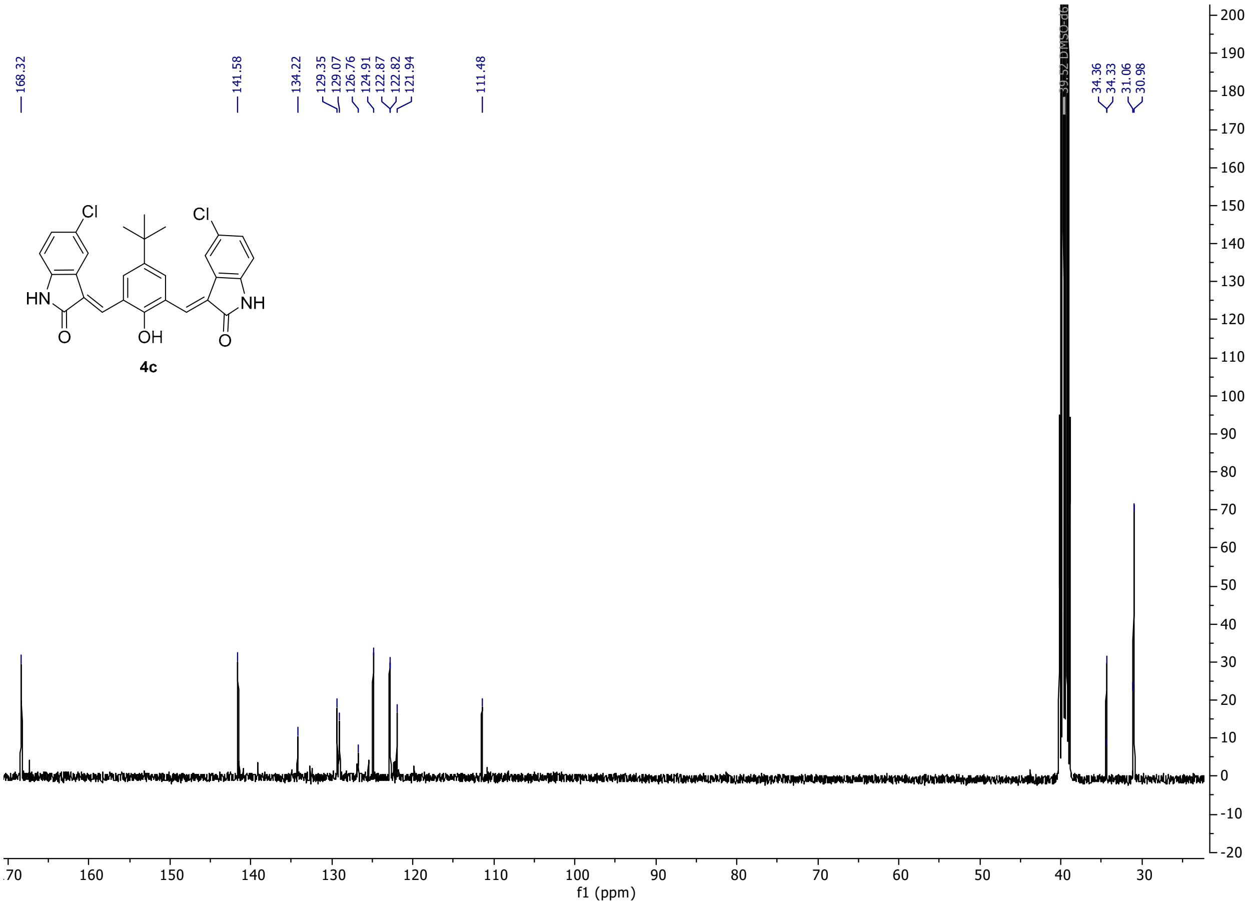
168.32
141.58
134.22
129.35
129.07
126.76
124.91
122.87
122.82
121.94
111.48
39.52 DMSO-d6
34.36
34.33
31.06
30.98
Cl
Cl
HN
NH
O
OH
O
4c
f1 (ppm)

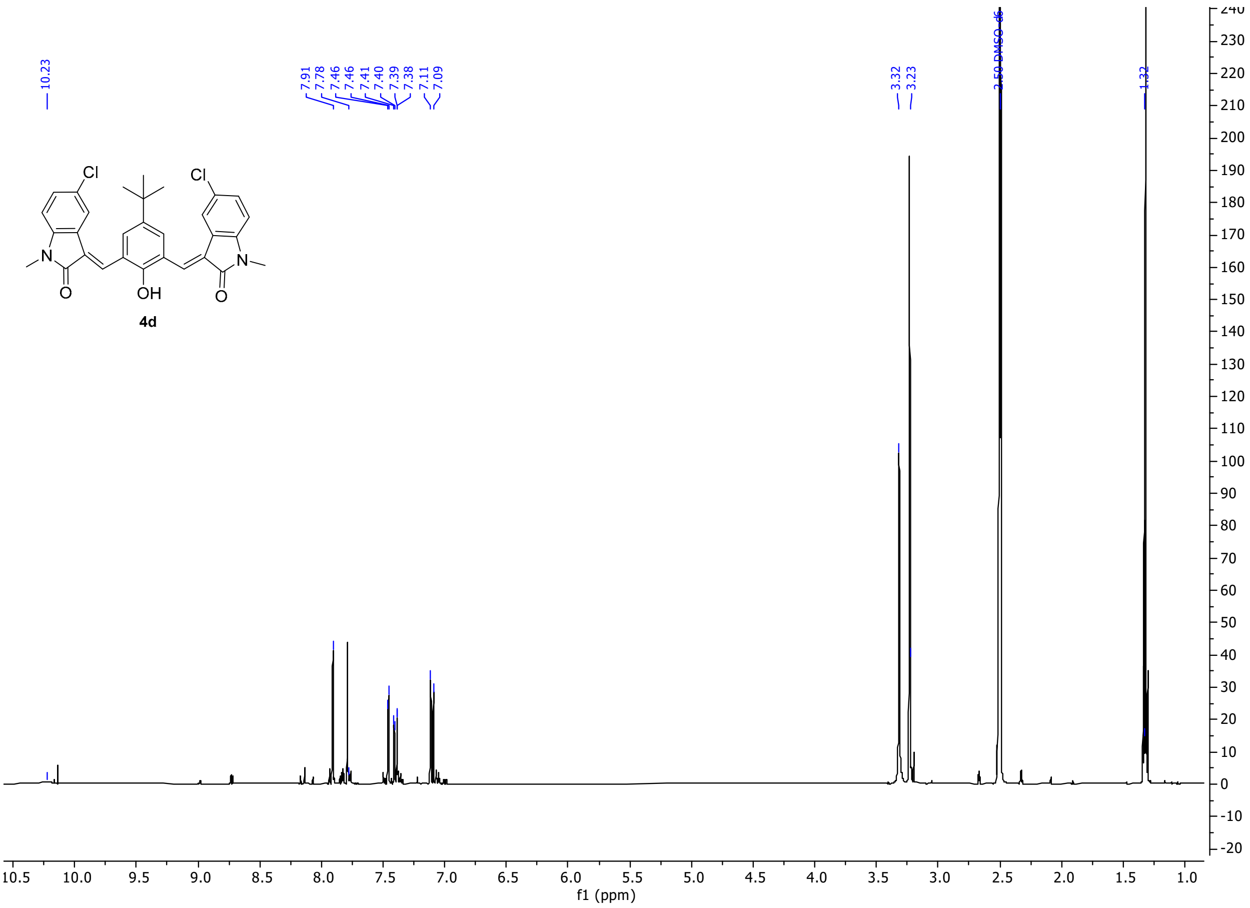
10.23
7.91
7.78
7.46
7.46
7.41
7.40
7.39
7.38
7.11
7.09
3.32
3.23
2.50 DMSO-d6
1.32
4d
f1 (ppm)

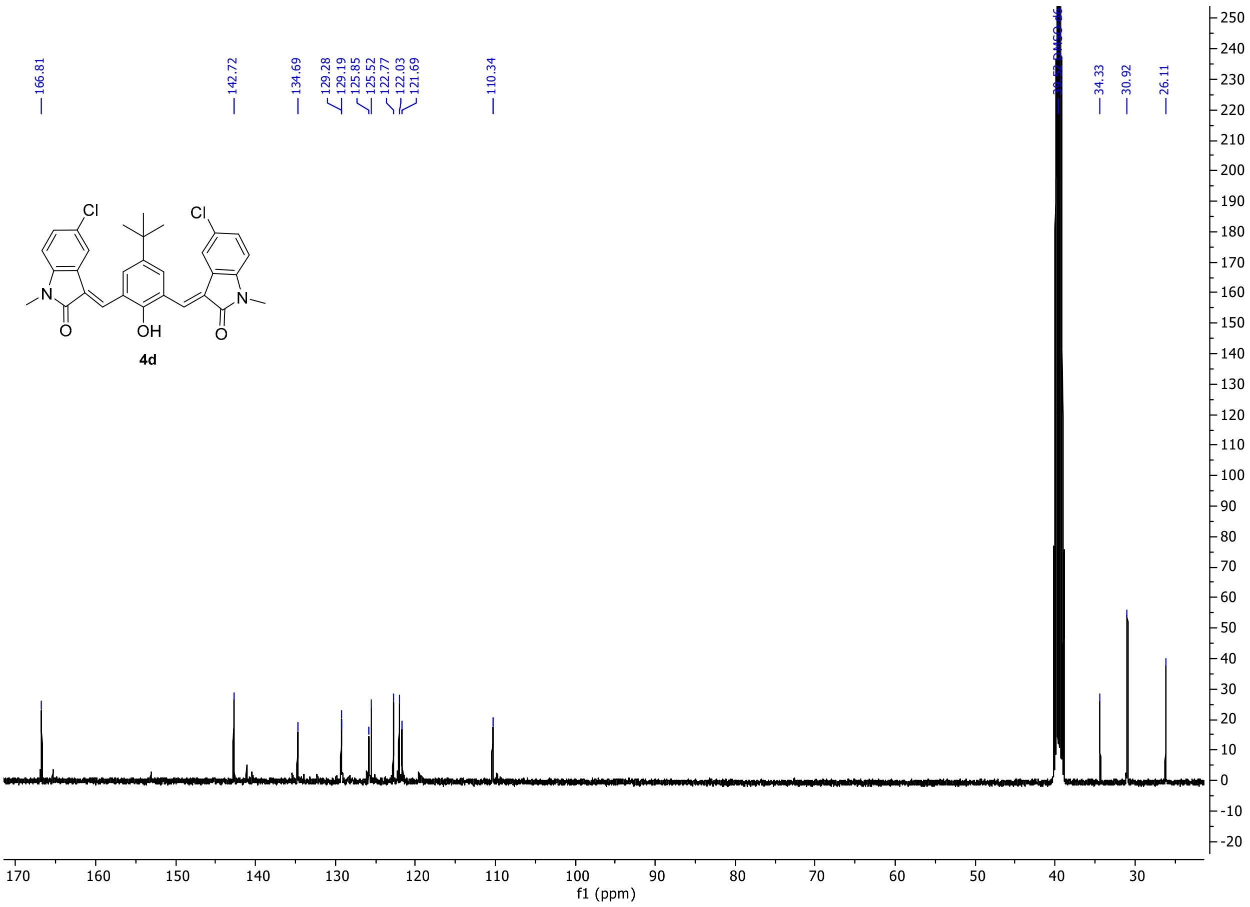
166.81
142.72
134.69
129.28
129.19
125.85
125.52
122.77
122.03
121.69
110.34
34.33
30.92
26.11
4d
f1 (ppm)

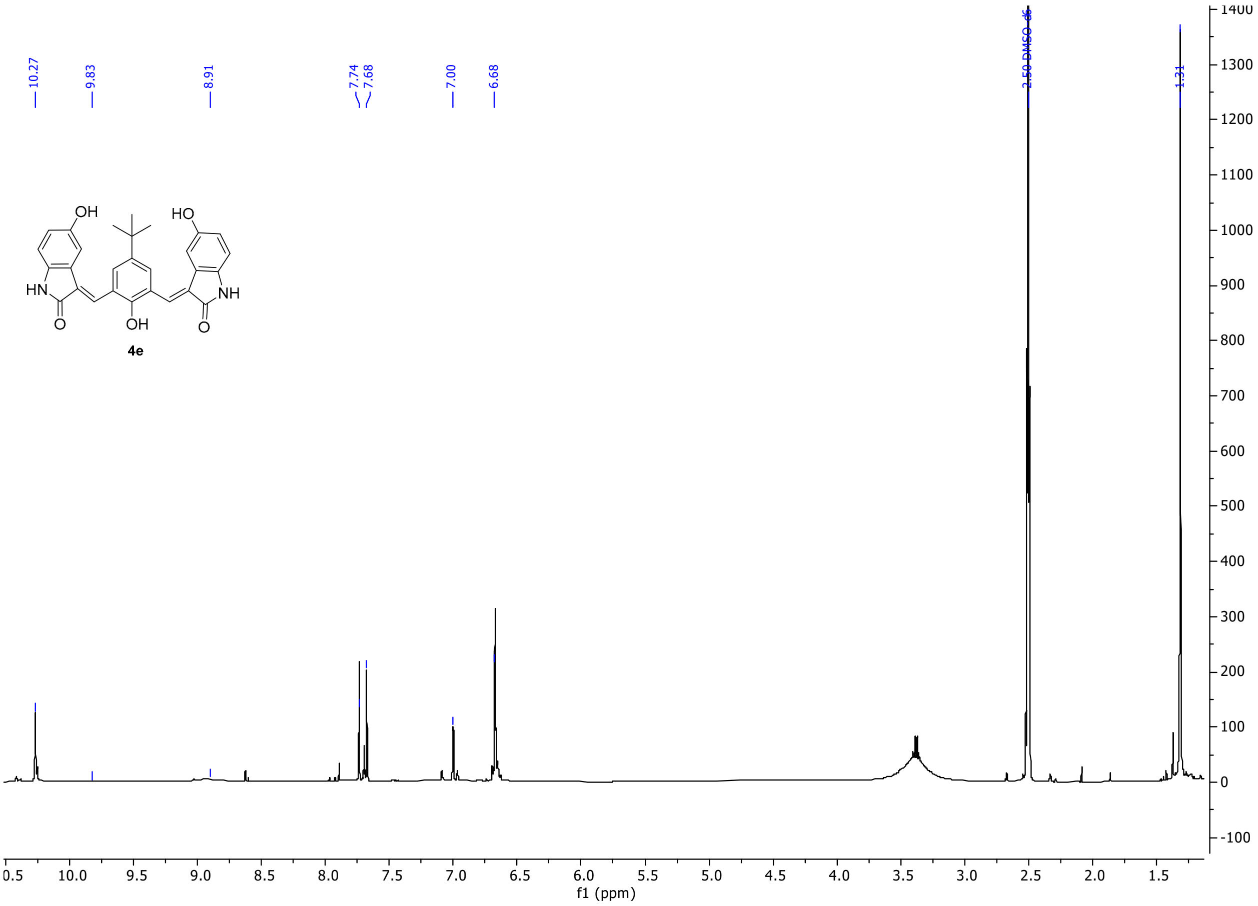
10.27
9.83
8.91
7.74
7.68
7.00
6.68
2.50 DMSO-d6
1.31
OH
HO
HN
NH
O
OH
4e
f1 (ppm)

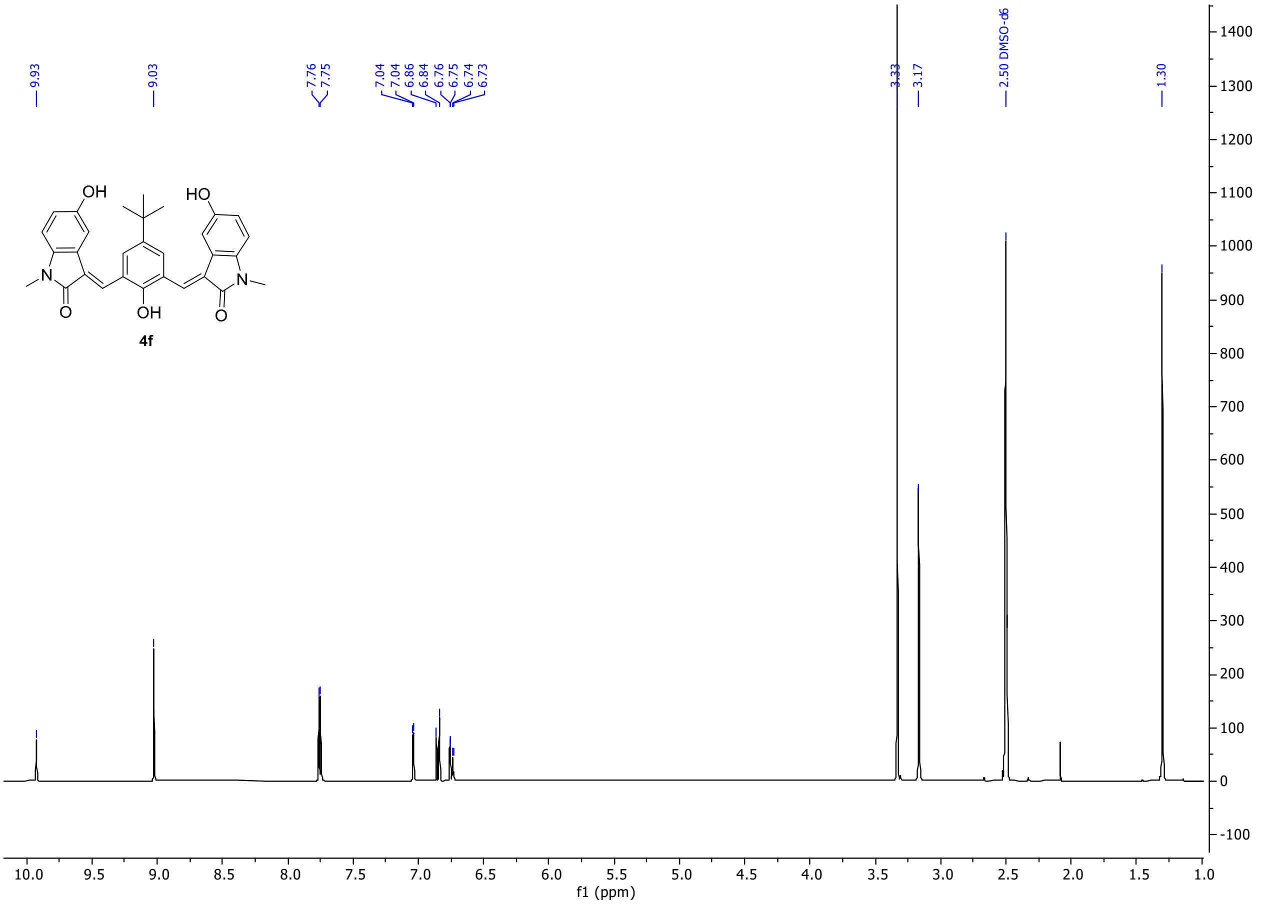
9.93
9.03
7.76
7.75
7.04
7.04
6.86
6.84
6.76
6.75
6.74
6.73
3.33
3.17
2.50 DMSO-d6
1.30
OH
HO
N
O
OH
4f
f1 (ppm)

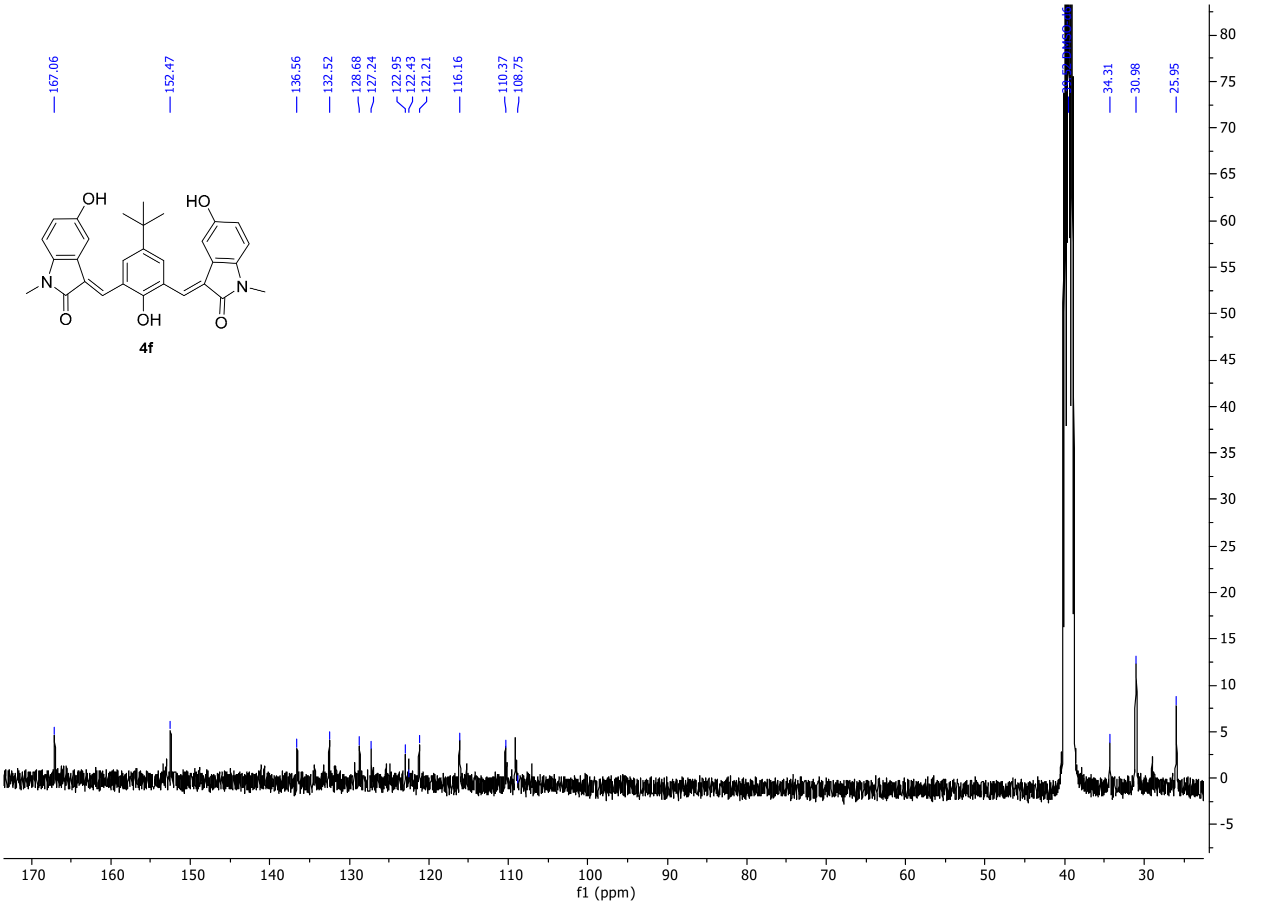
167.06
152.47
136.56
132.52
128.68
127.24
122.95
122.43
121.21
116.16
110.37
108.75
34.31
30.98
25.95
OH
HO
N
O
OH
4f
f1 (ppm)

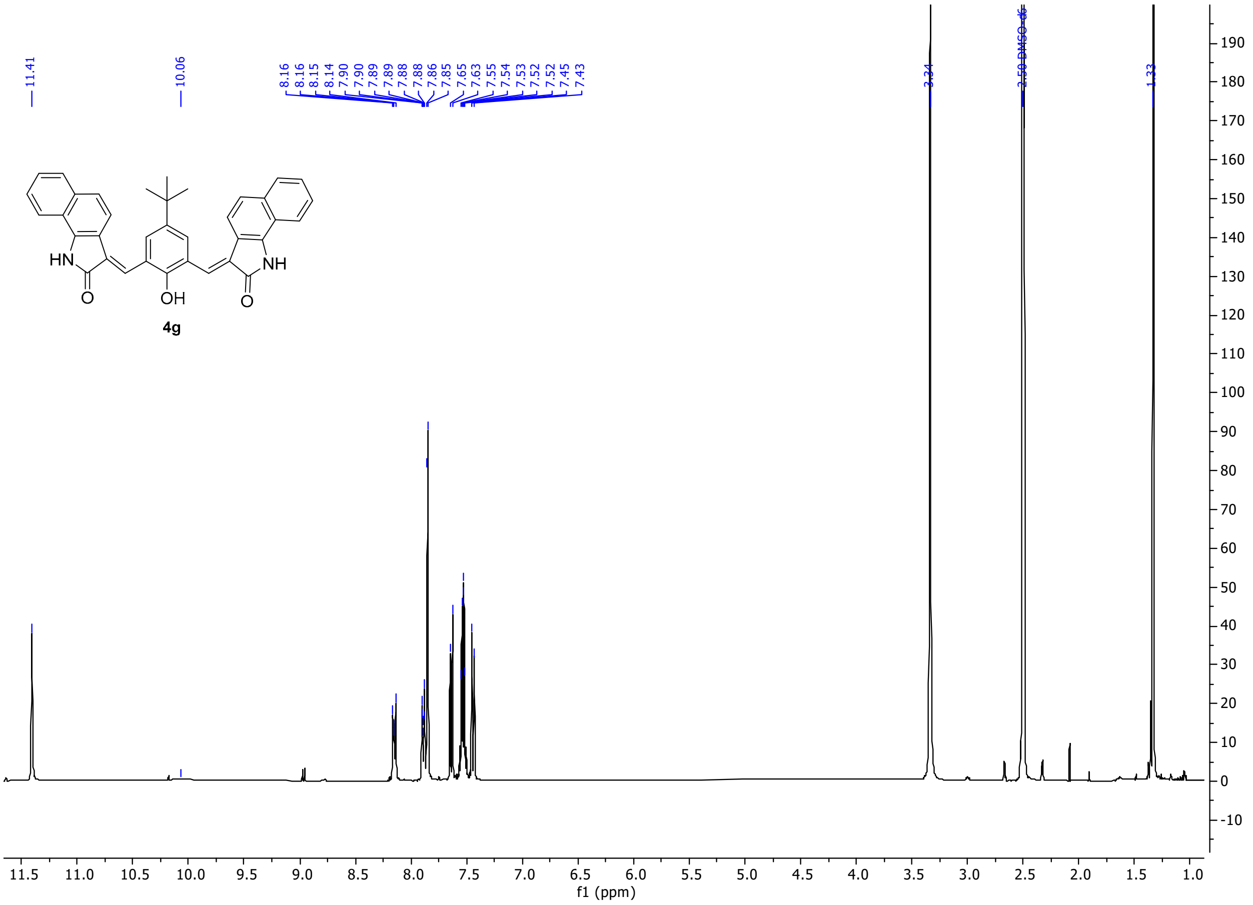
11.41
10.06
8.16 8.16 8.15 8.14 7.90 7.90 7.89 7.89 7.88 7.88 7.86 7.85 7.65 7.63 7.55 7.54 7.53 7.52 7.52 7.45 7.43
3.34
2.50 DMSO-d6
1.33
HN
NH
OH
O
4g
f1 (ppm)

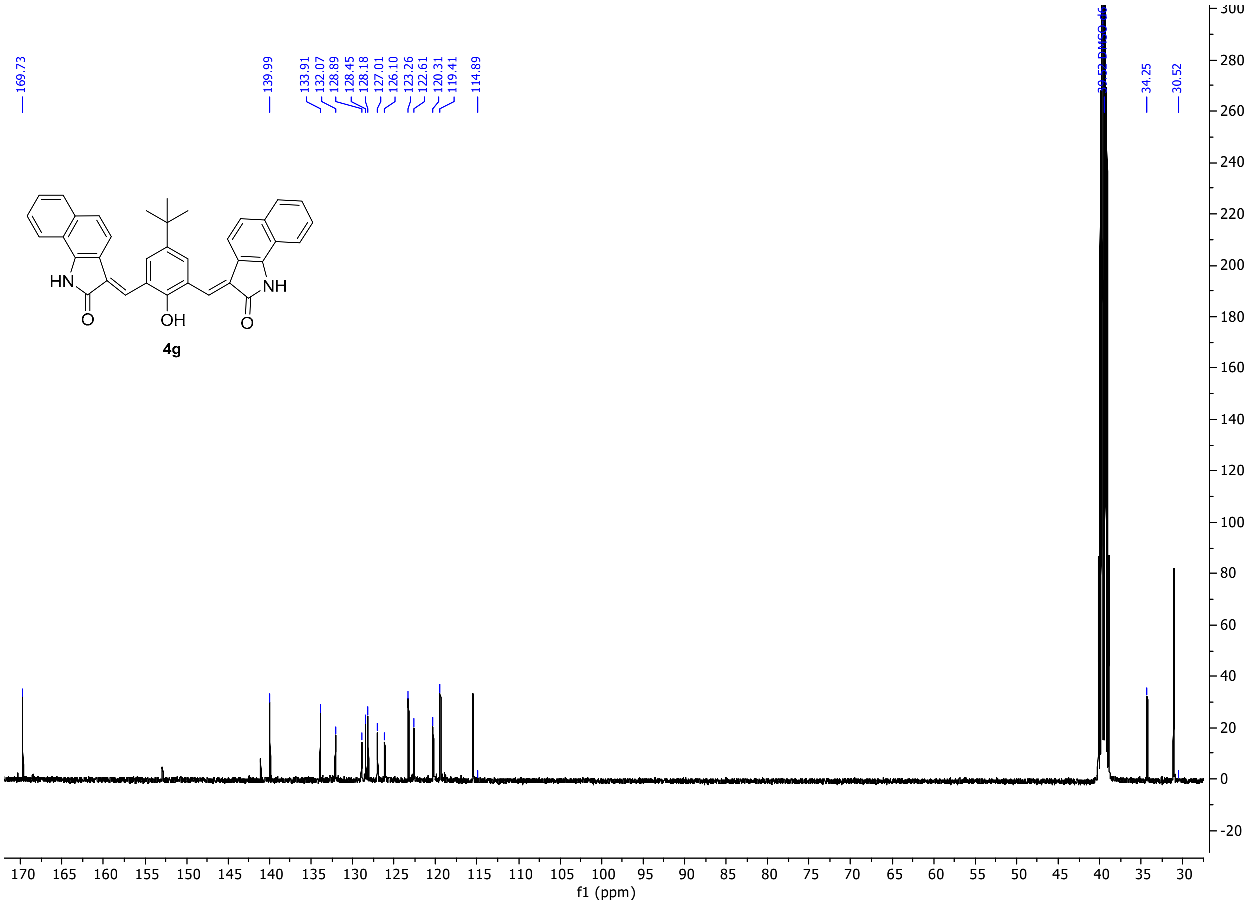
169.73
139.99
133.91 132.07 128.89 128.45 128.18 127.01 126.10 123.26 122.61 120.31 119.41
114.89
39.52 DMSO-d6
34.25
30.52
HN
NH
OH
O
4g
f1 (ppm)

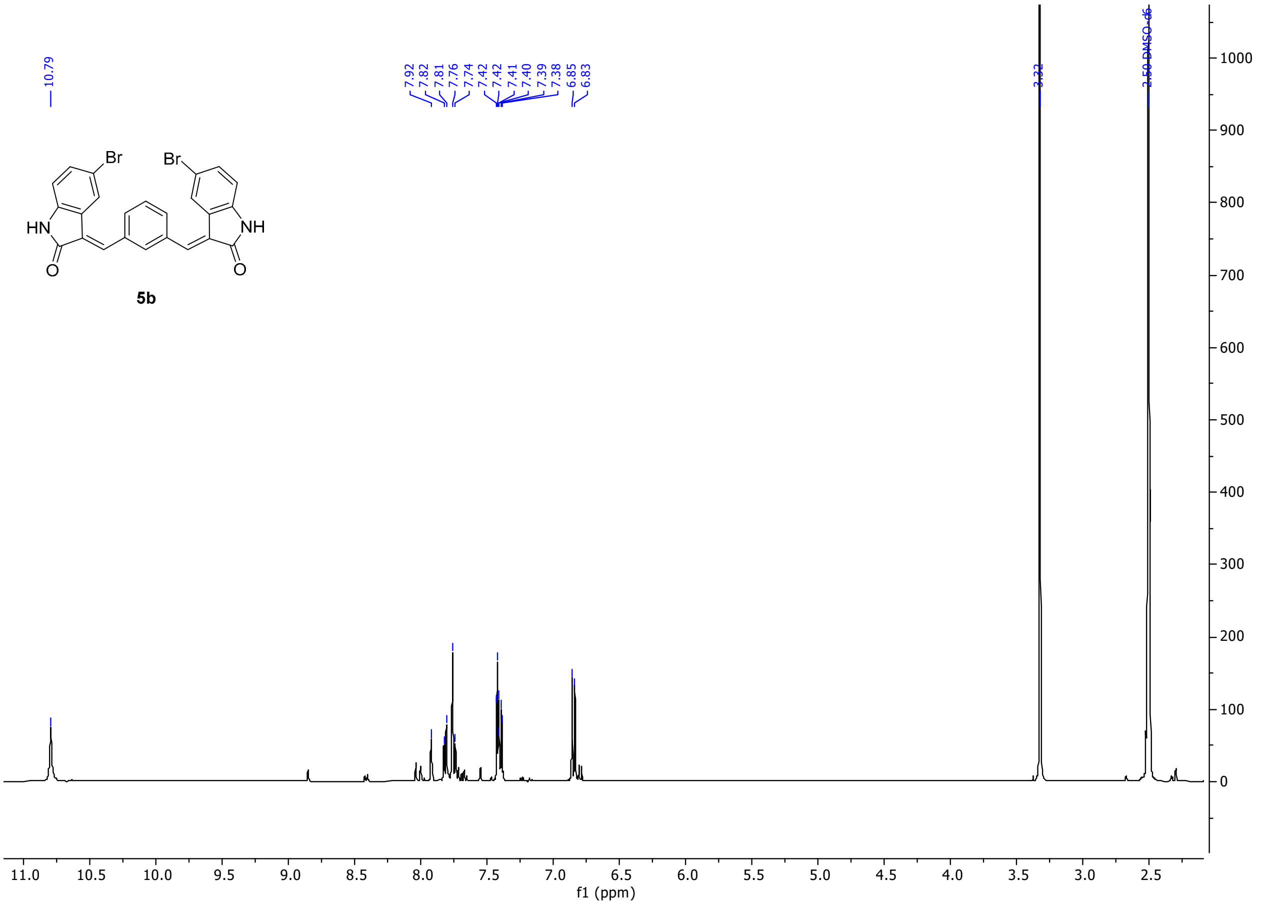
10.79
7.92
7.82
7.81
7.76
7.74
7.42
7.42
7.41
7.40
7.39
7.38
6.85
6.83
3.32
2.50 DMSO-d6
Br
Br
HN
NH
O
O
5b
f1 (ppm)

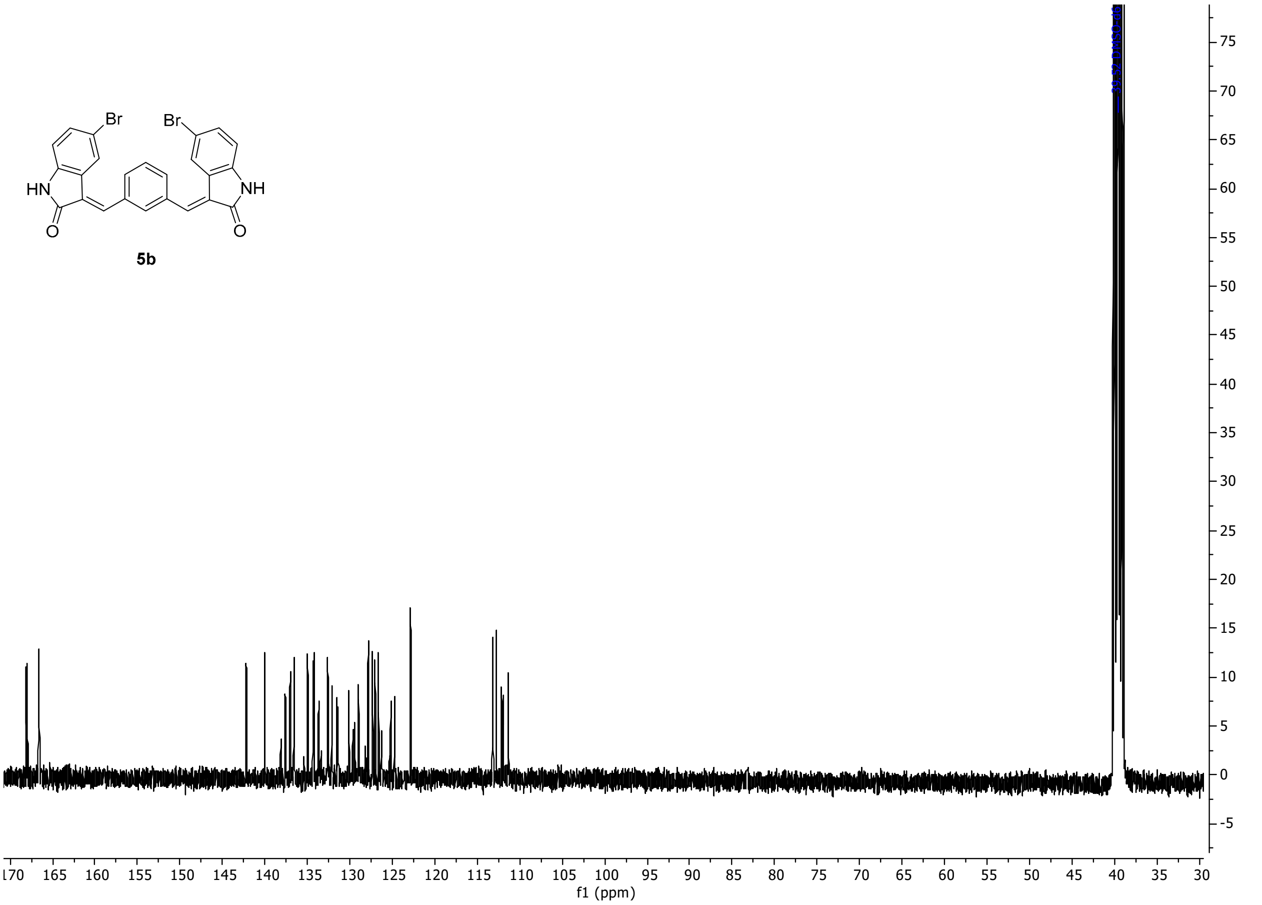
Br
Br
HN
NH
O
O
5b
f1 (ppm)

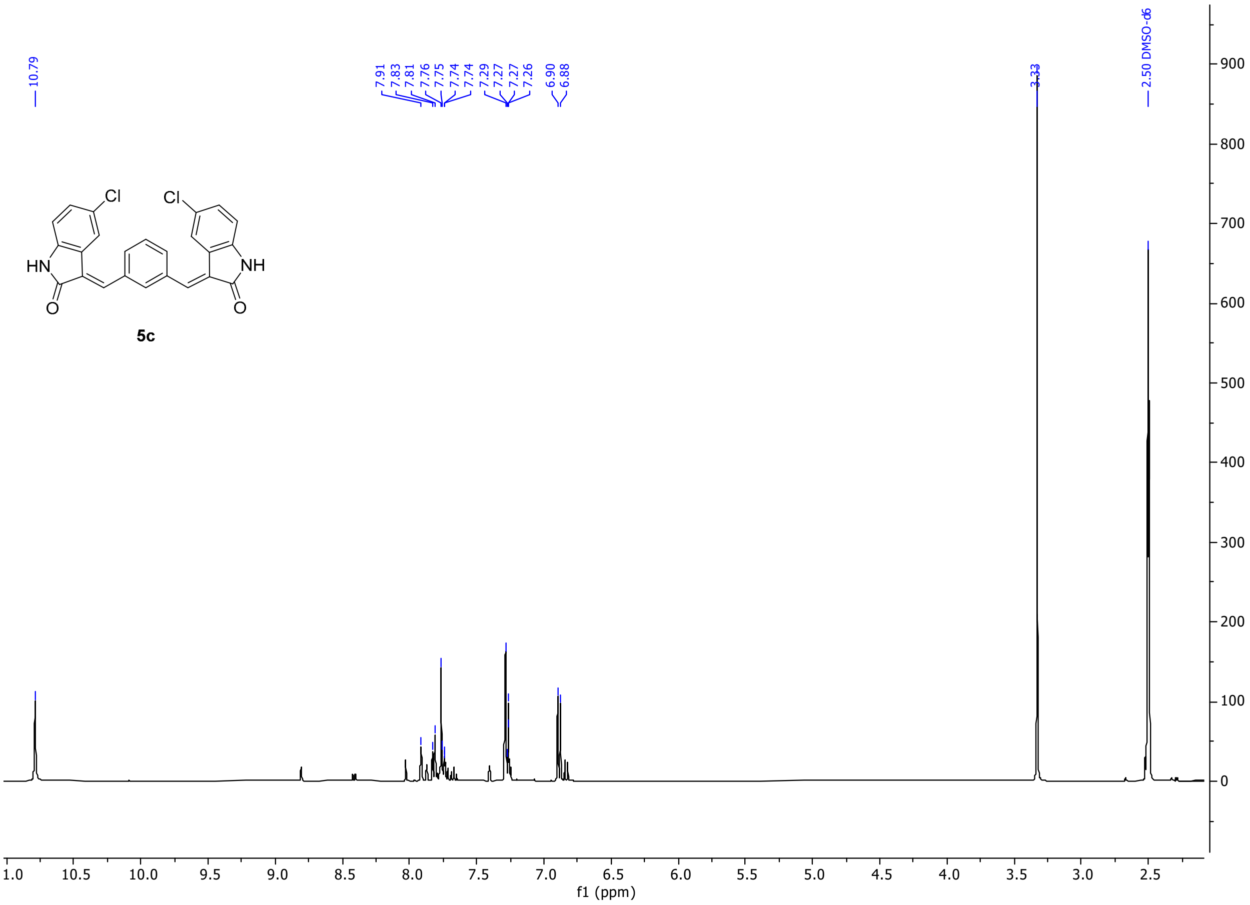
10.79
7.91
7.83
7.81
7.76
7.75
7.74
7.74
7.29
7.27
7.27
7.26
6.90
6.88
3.33
2.50 DMSO-d6
Cl
Cl
HN
NH
O
O
5c
f1 (ppm)

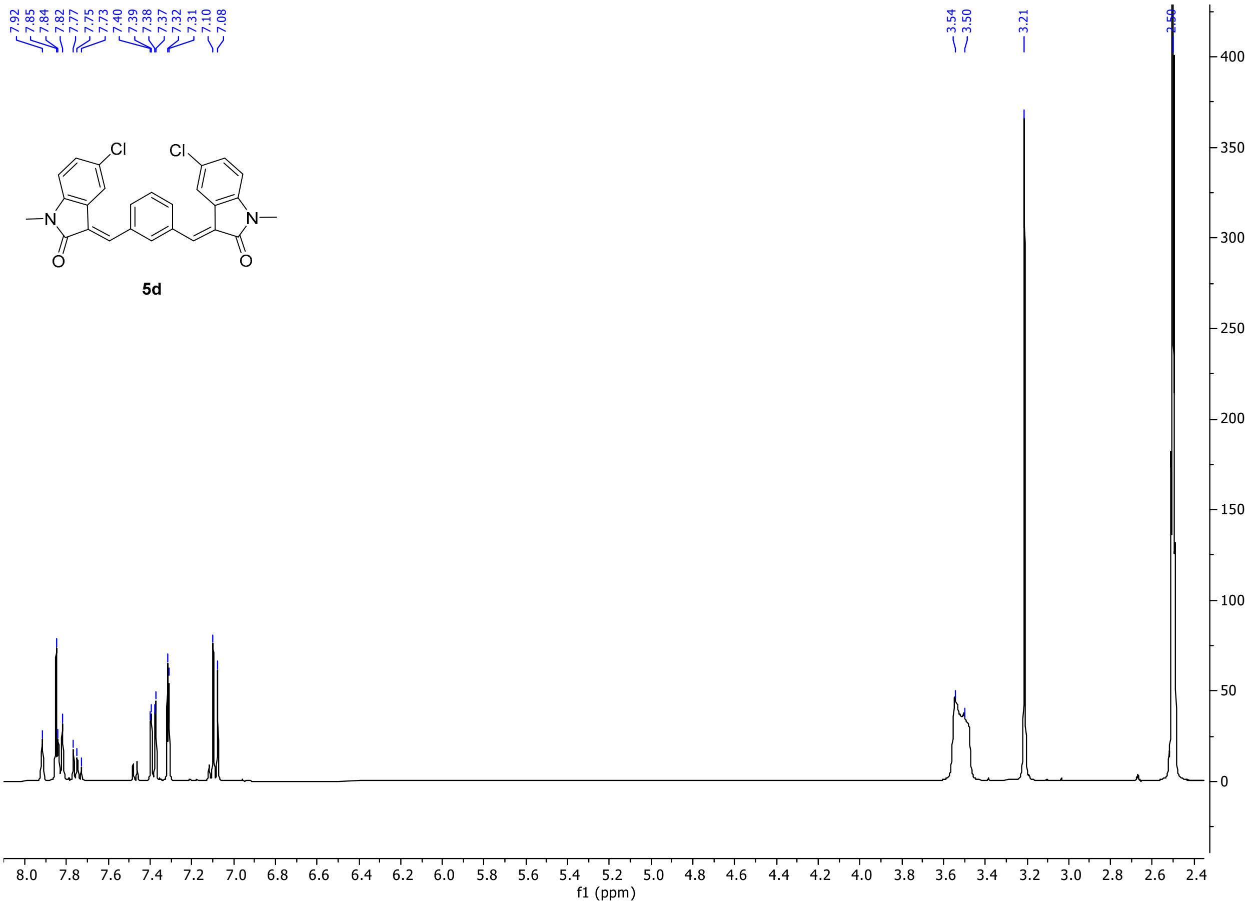
7.92
7.85
7.84
7.82
7.77
7.75
7.73
7.40
7.39
7.38
7.37
7.32
7.31
7.10
7.08
3.54
3.50
3.21
2.50
Cl
Cl
N
N
O
O
5d
f1 (ppm)

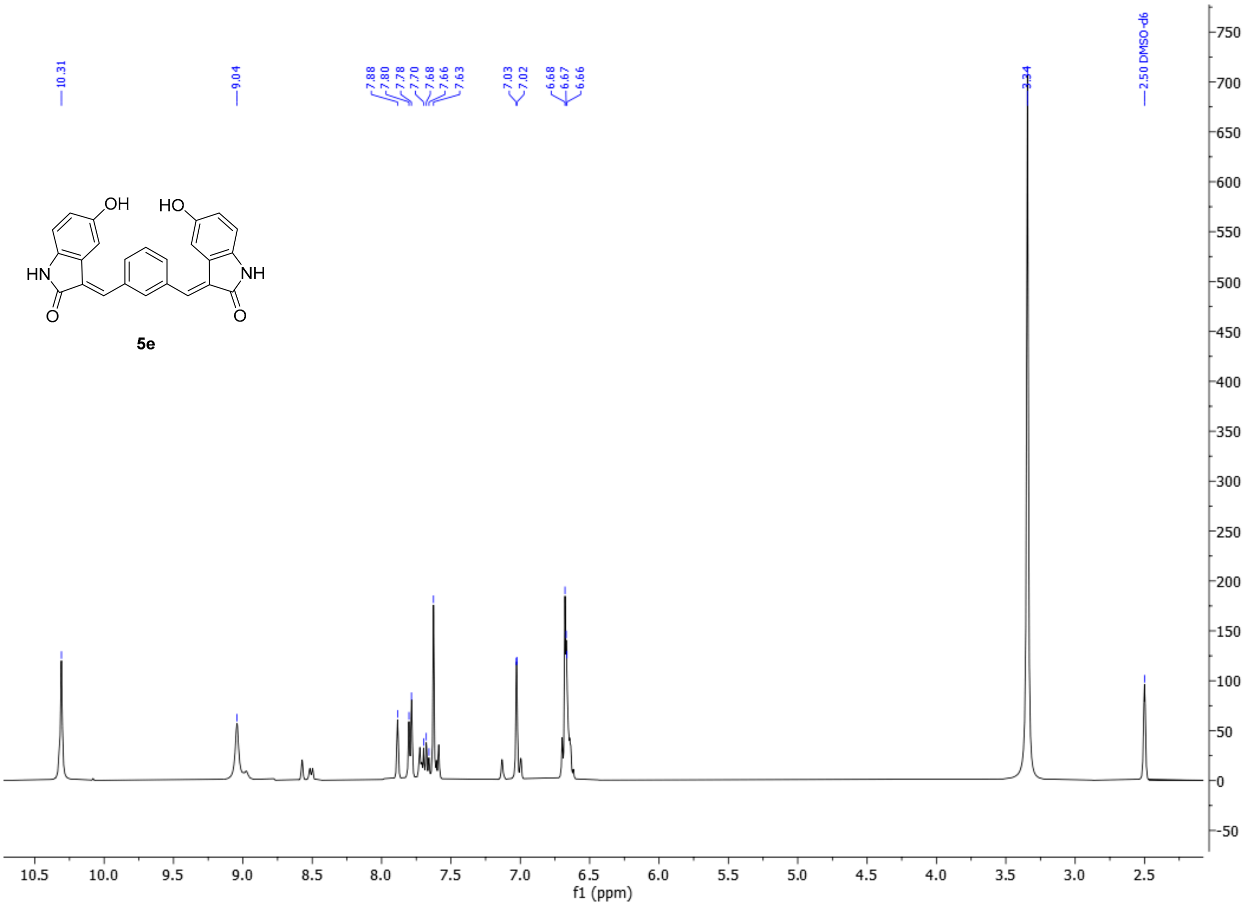
10.31
9.04
7.88
7.80
7.78
7.70
7.68
7.66
7.63
7.03
7.02
6.68
6.67
6.66
3.34
2.50 DMSO-d6
5e
f1 (ppm)

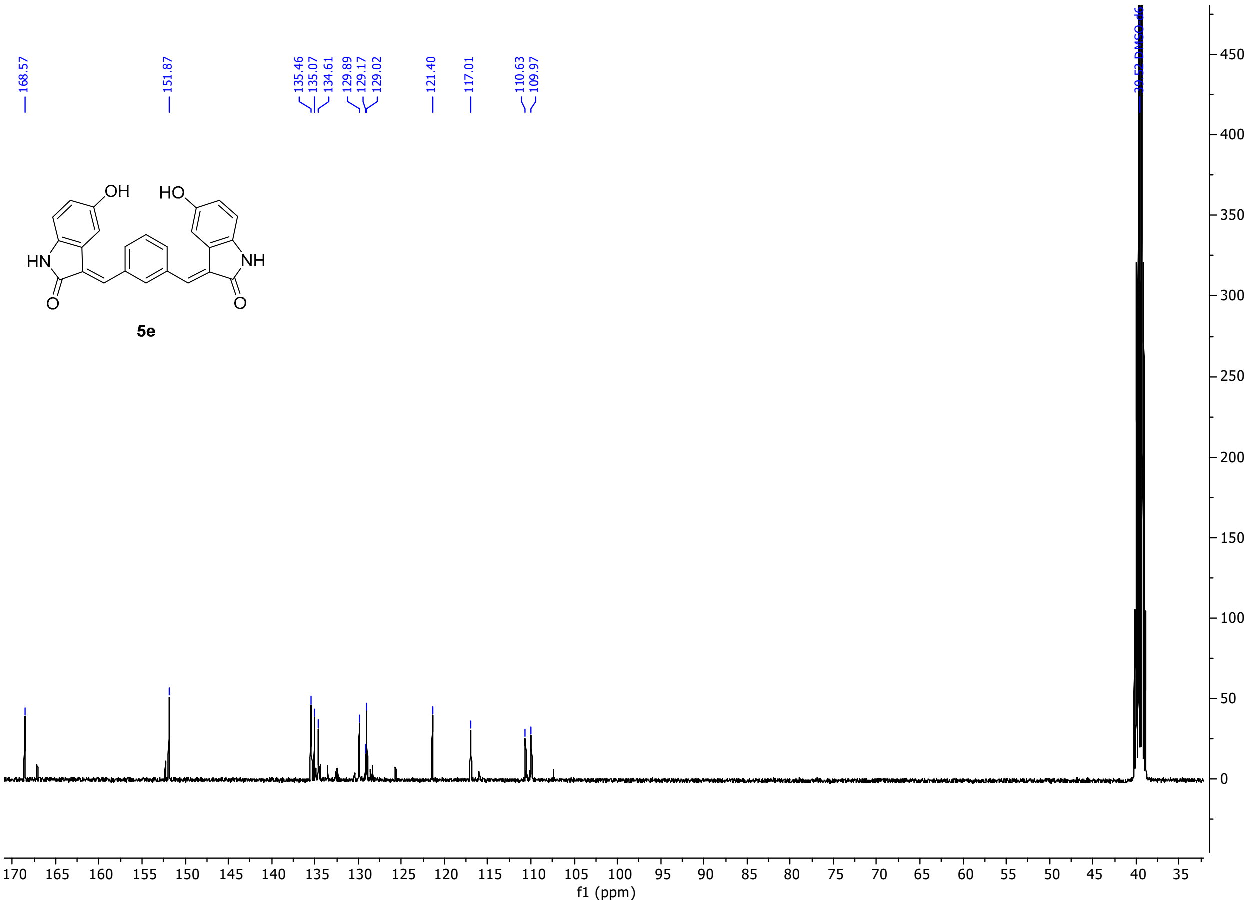
168.57
151.87
135.46
135.07
134.61
129.89
129.17
129.02
121.40
117.01
110.63
109.97
5e
f1 (ppm)

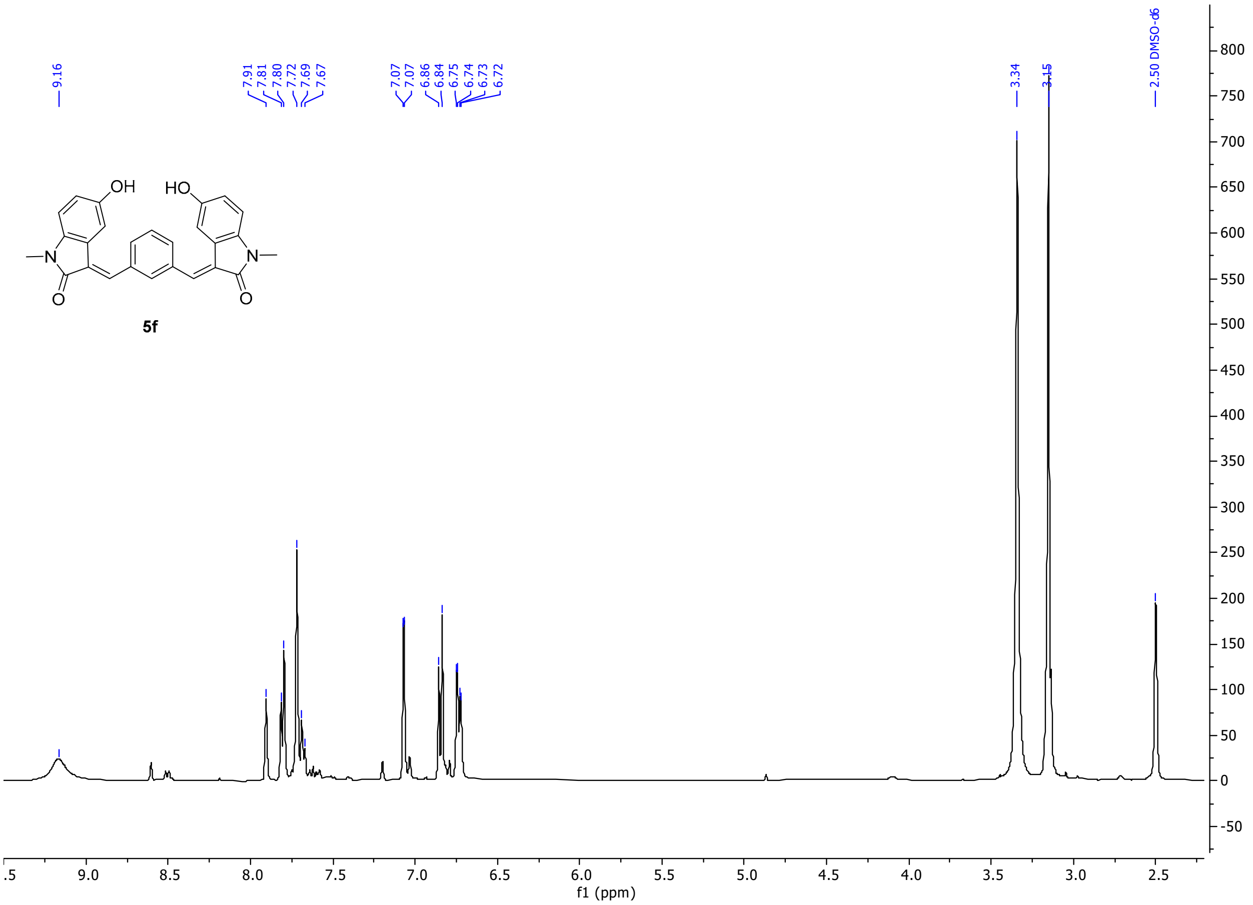

9.16
7.91
7.81
7.80
7.72
7.69
7.67
7.07
7.07
6.86
6.84
6.75
6.74
6.73
6.72
3.34
3.15
2.50 DMSO-d6
OH
HO
N
O
5f
f1 (ppm)

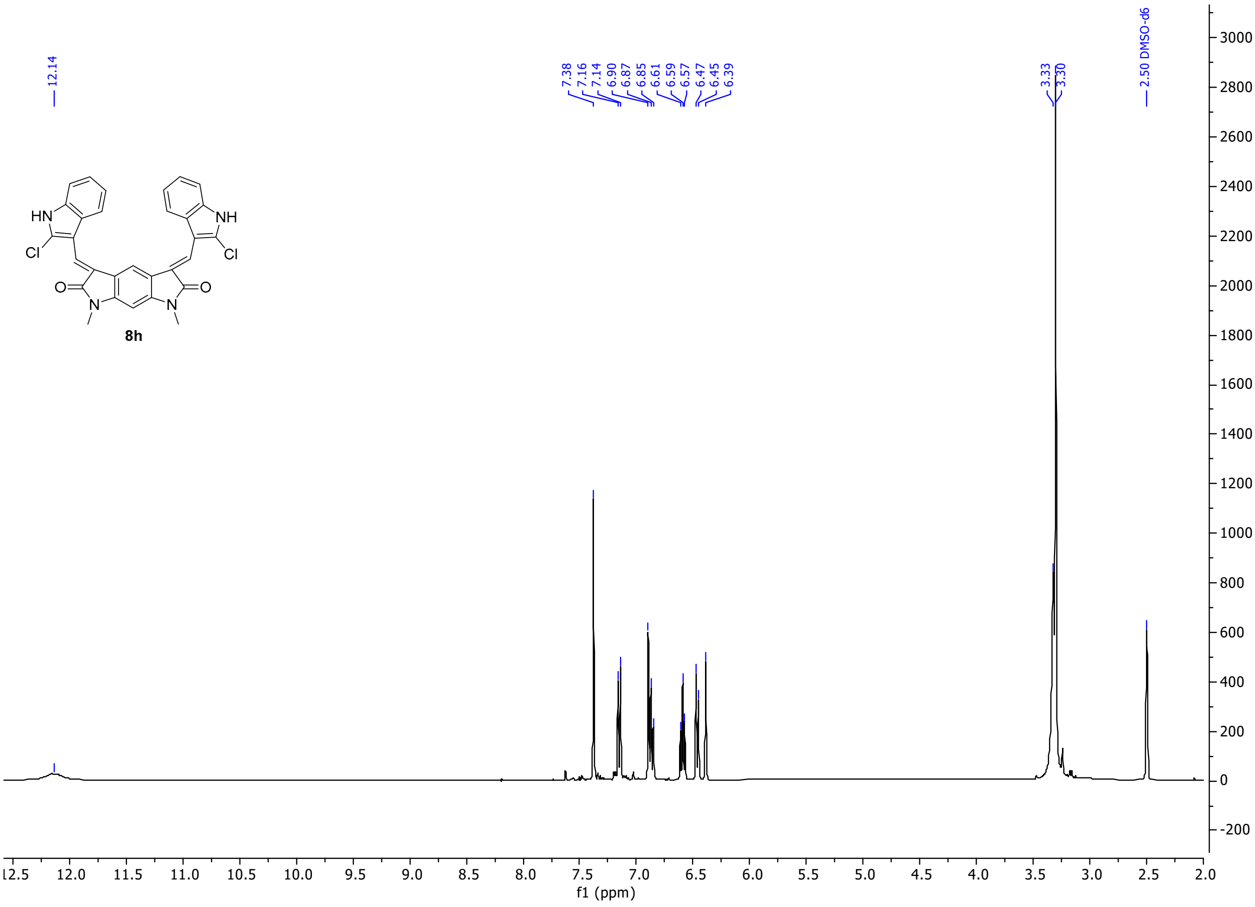

12.14
7.38
7.16
7.14
6.90
6.87
6.85
6.61
6.59
6.57
6.47
6.45
6.39
3.33
3.30
2.50 DMSO-d6
HN
NH
Cl
O
N
8h
f1 (ppm)

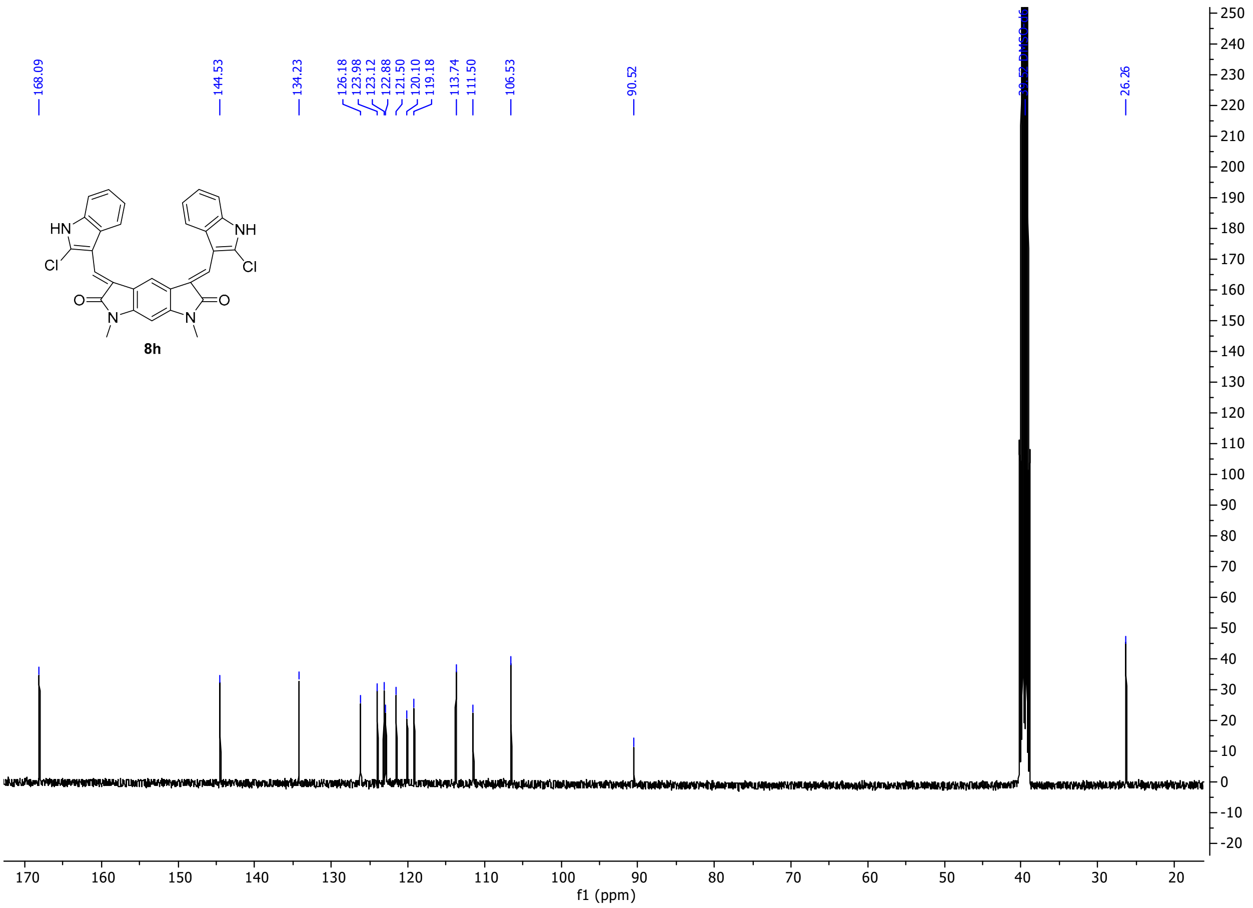
168.09
144.53
134.23
126.18
123.98
123.12
122.88
121.50
120.10
119.18
113.74
111.50
106.53
90.52
26.26
HN
NH
Cl
Cl
O
O
N
N
8h
f1 (ppm)
170 160 150 140 130 120 110 100 90 80 70 60 50 40 30 20
250 240 230 220 210 200 190 180 170 160 150 140 130 120 110 100 90 80 70 60 50 40 30 20 10 0 -10 -20

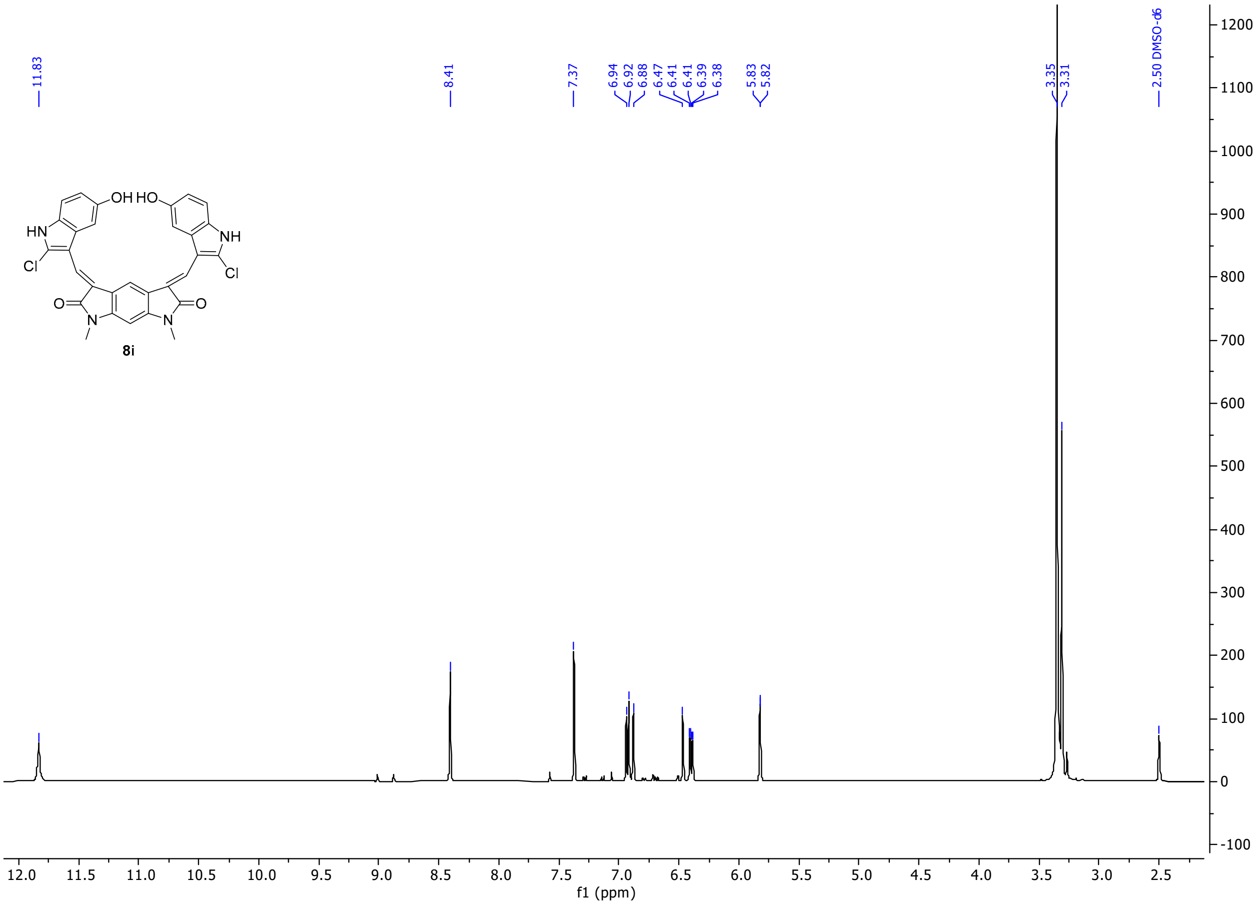
11.83
8.41
7.37
6.94
6.92
6.88
6.47
6.41
6.41
6.39
6.38
5.83
5.82
3.35
3.31
2.50 DMSO-d6
OH HO
HN
NH
Cl
Cl
O
O
N
N
8i
f1 (ppm)

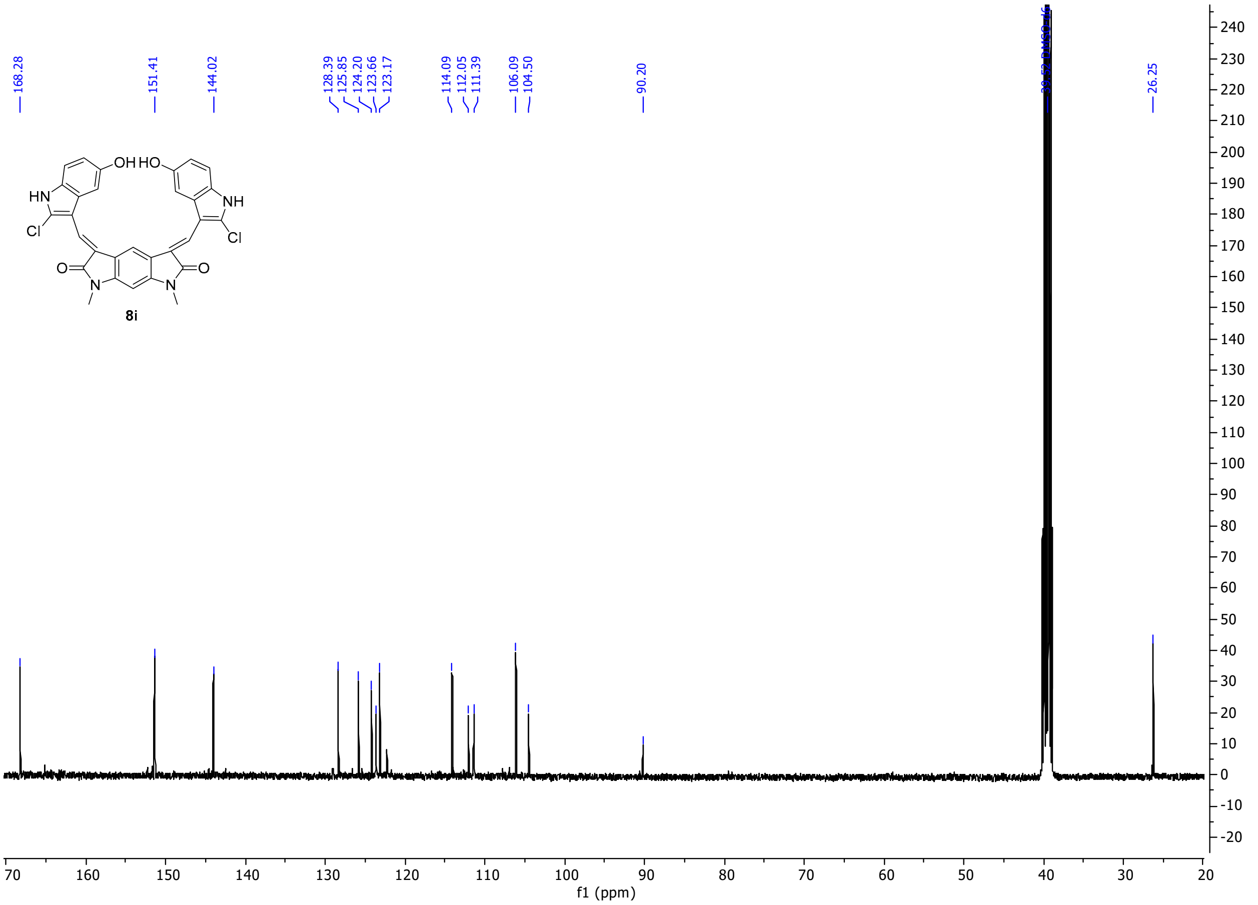
168.28
151.41
144.02
128.39
125.85
124.20
123.66
123.17
114.09
112.05
111.39
106.09
104.50
90.20
26.25
OH HO
HN
NH
Cl
Cl
O
O
N
N
8i
f1 (ppm)

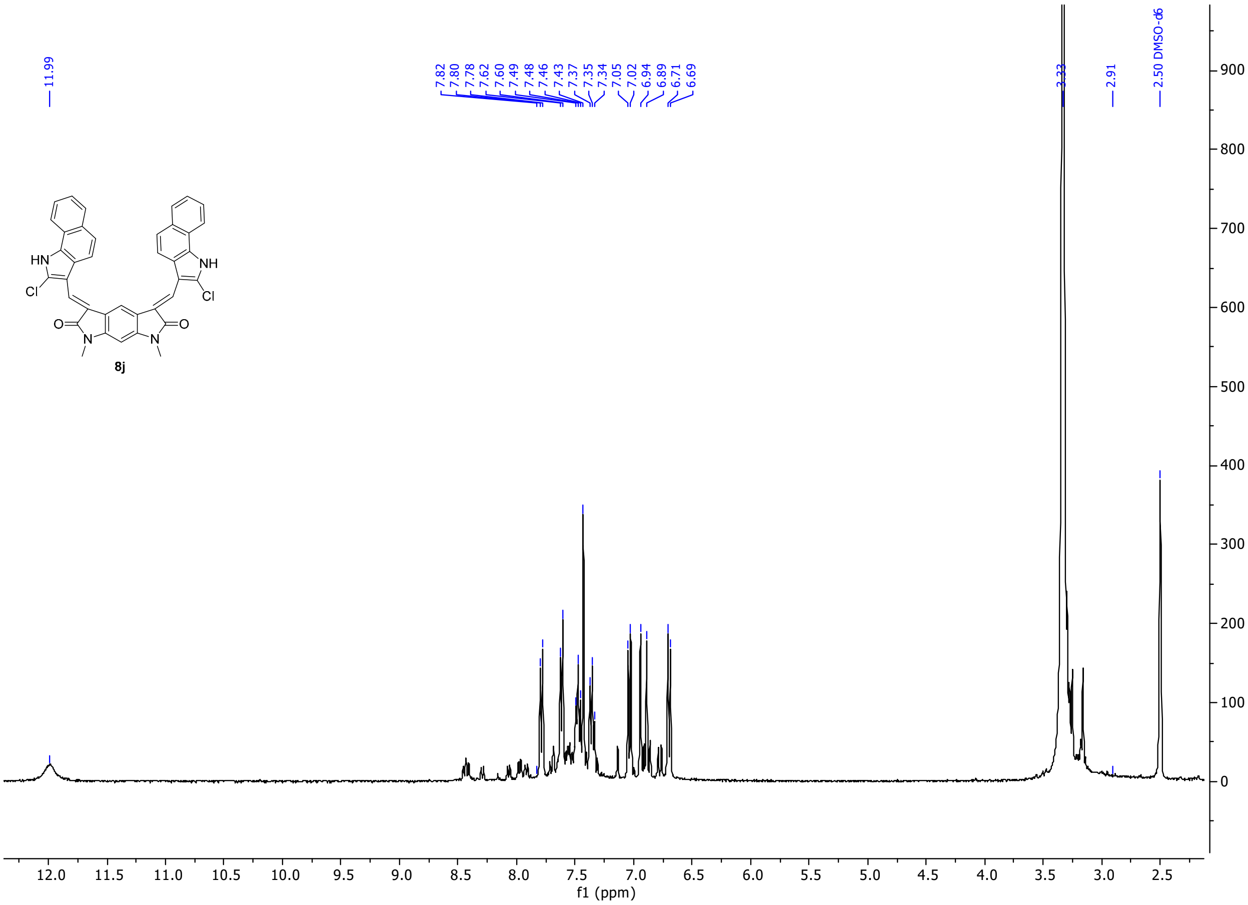
11.99
7.82 7.80 7.78 7.62 7.60 7.49 7.48 7.46 7.43 7.37 7.35 7.34 7.05 7.02 6.94 6.89 6.71 6.69
3.33
2.91
2.50 DMSO-d6
HN
NH
Cl
O
N
8j
f1 (ppm)

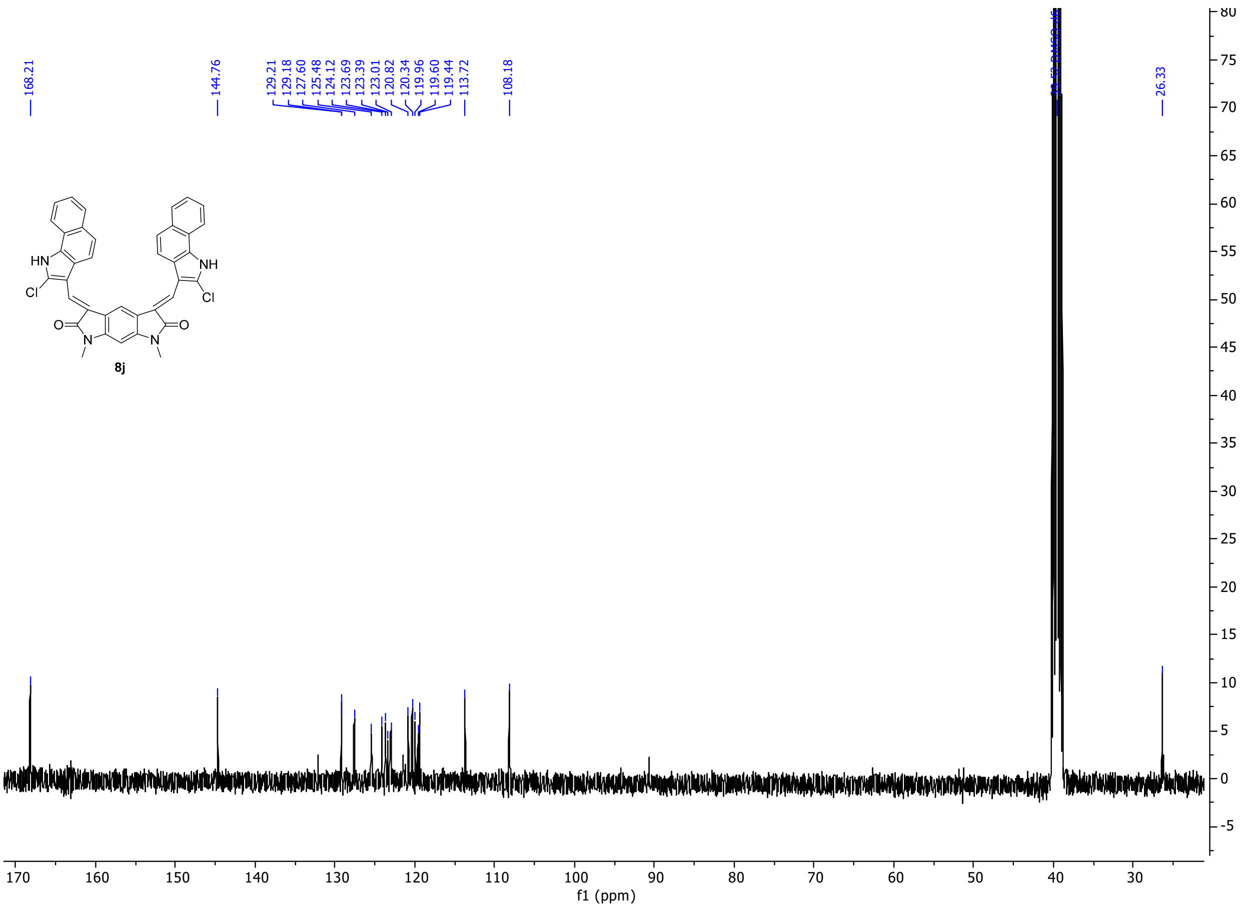
168.21
144.76
129.21 129.18 127.60 125.48 124.12 123.69 123.39 123.01 120.82 120.34 119.96 119.60 119.44 113.72
108.18
26.33
HN
NH
Cl
O
N
8j
f1 (ppm)

HRMS spectra of the described compounds

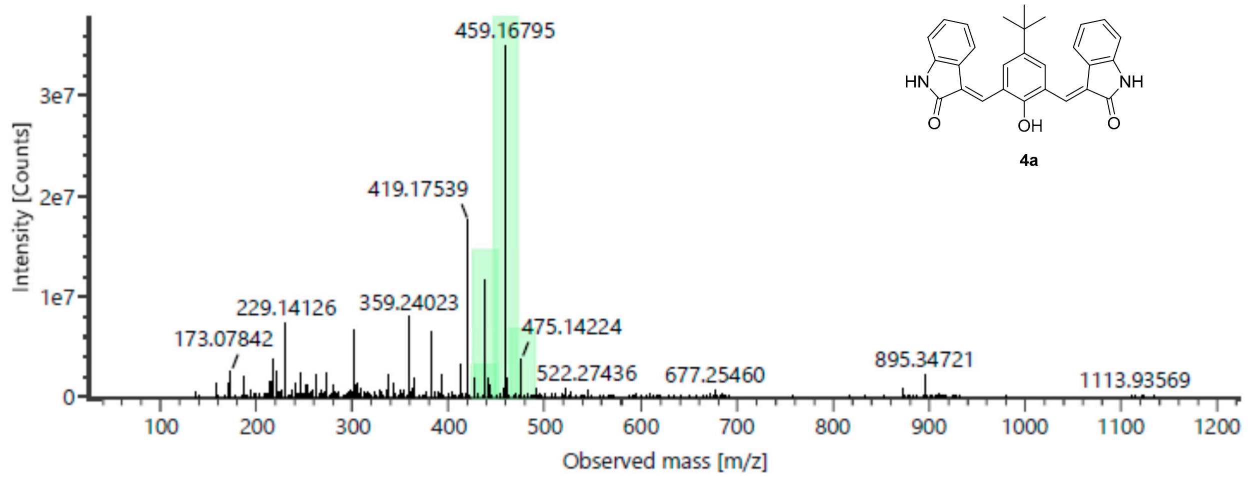

4a

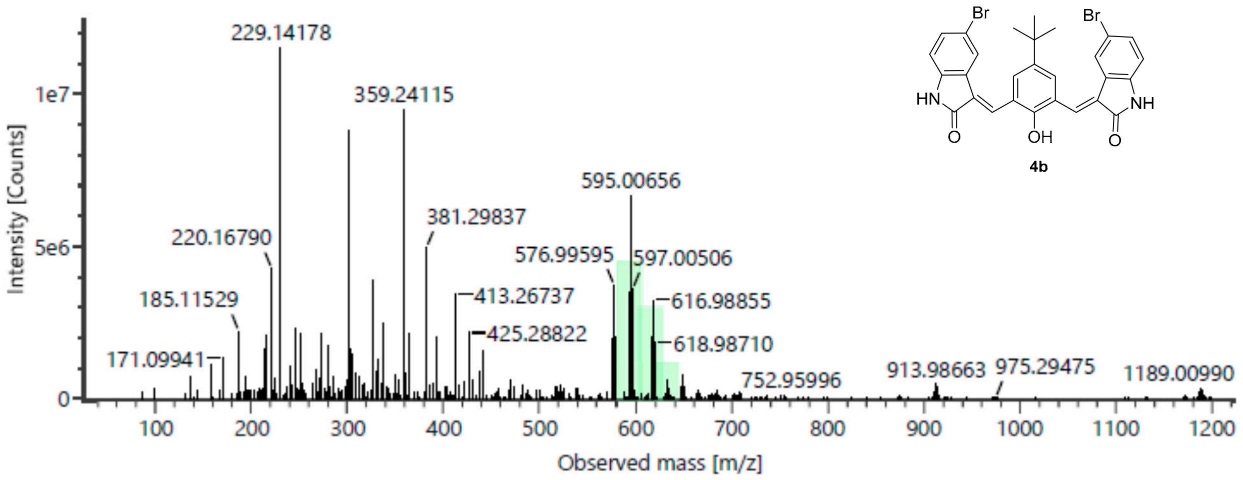

4b

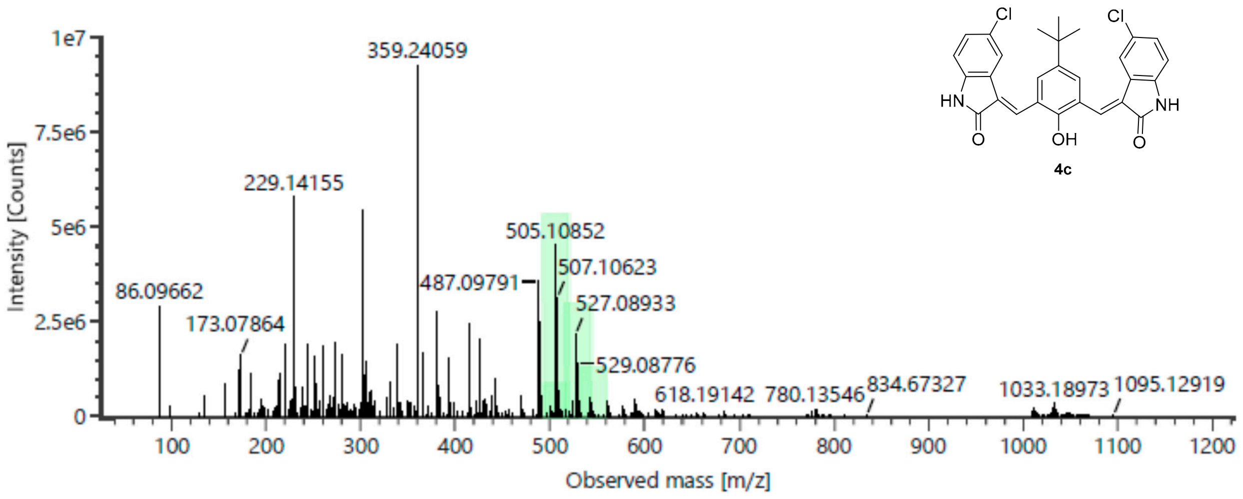

4c

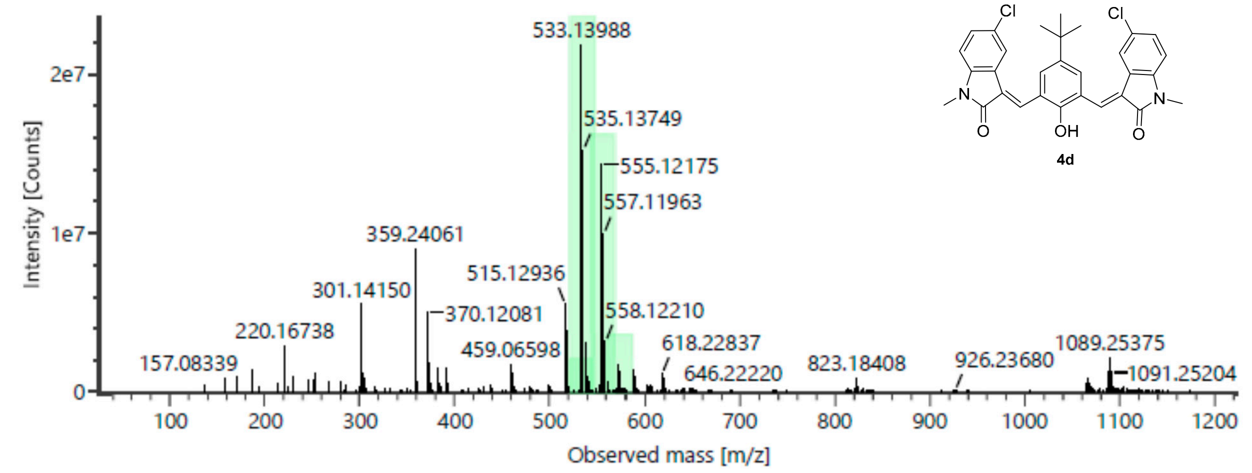
533.13988
535.13749
555.12175
557.11963
359.24061
301.14150
515.12936
370.12081
220.16738
558.12210
459.06598
157.08339
618.22837
646.22220
823.18408
926.23680
1089.25375
1091.25204
Intensity [Counts]
Observed mass [m/z]
4d

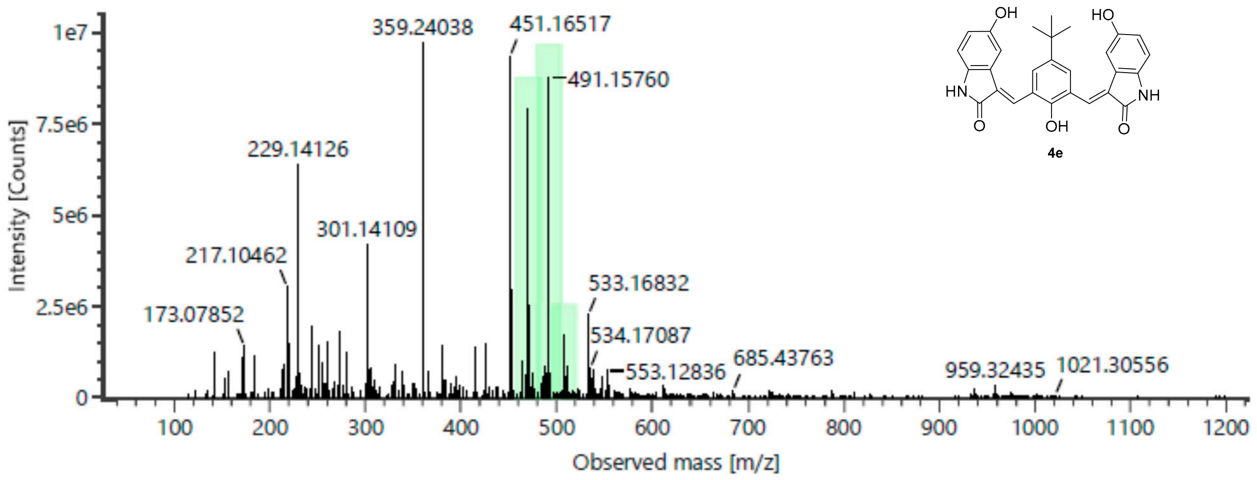
359.24038
451.16517
491.15760
229.14126
301.14109
217.10462
533.16832
173.07852
534.17087
553.12836
685.43763
959.32435
1021.30556
Intensity [Counts]
Observed mass [m/z]
4e

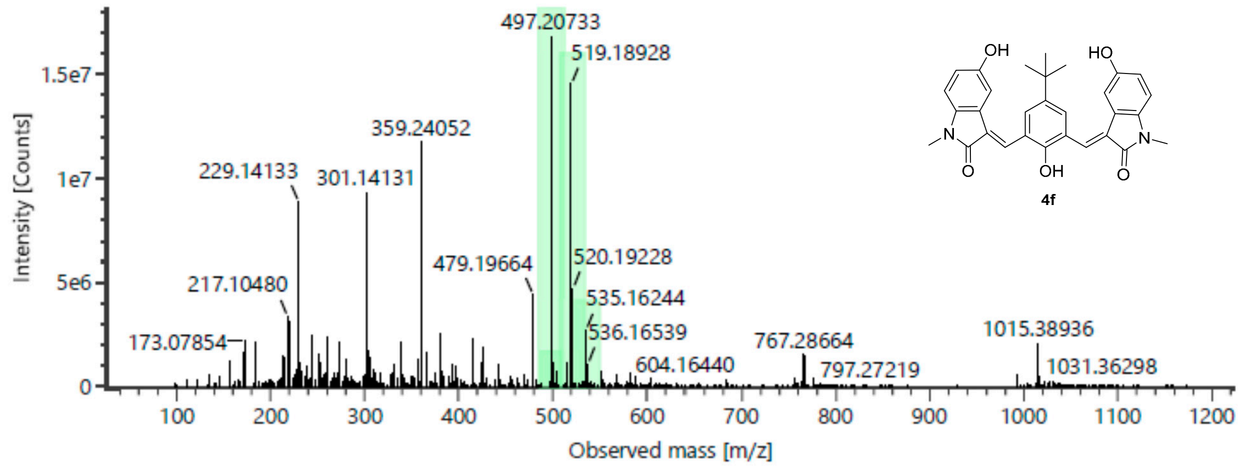
497.20733
519.18928
359.24052
229.14133
301.14131
479.19664
520.19228
217.10480
535.16244
536.16539
173.07854
767.28664
604.16440
797.27219
1015.38936
1031.36298
Intensity [Counts]
Observed mass [m/z]
4f

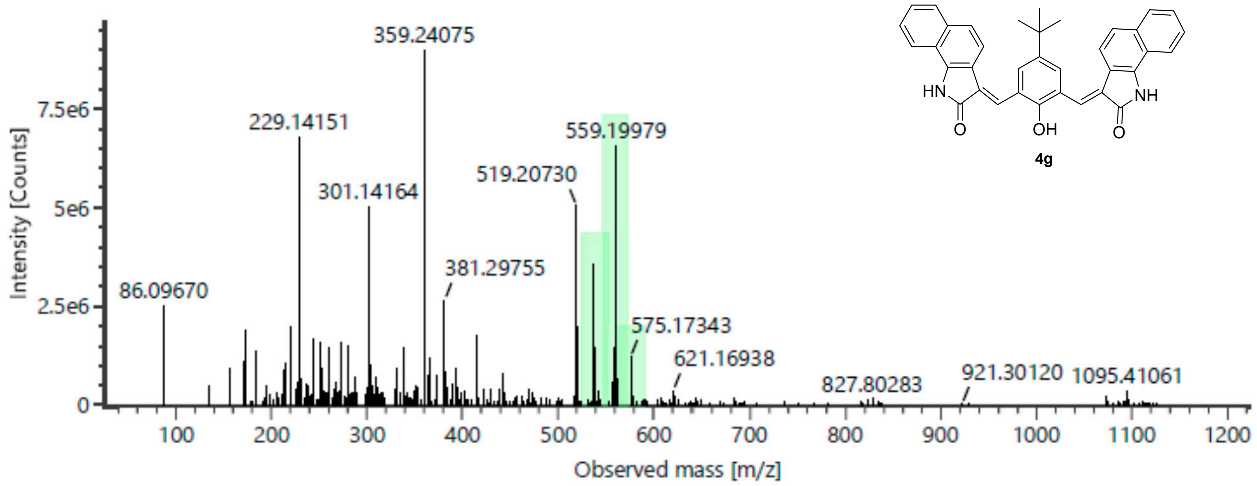
359.24075
229.14151
559.19979
7.5e6
5e6
2.5e6
0
301.14164
519.20730
381.29755
86.09670
575.17343
621.16938
827.80283
921.30120
1095.41061
Intensity [Counts]
Observed mass [m/z]
HN
NH
O
OH
4g

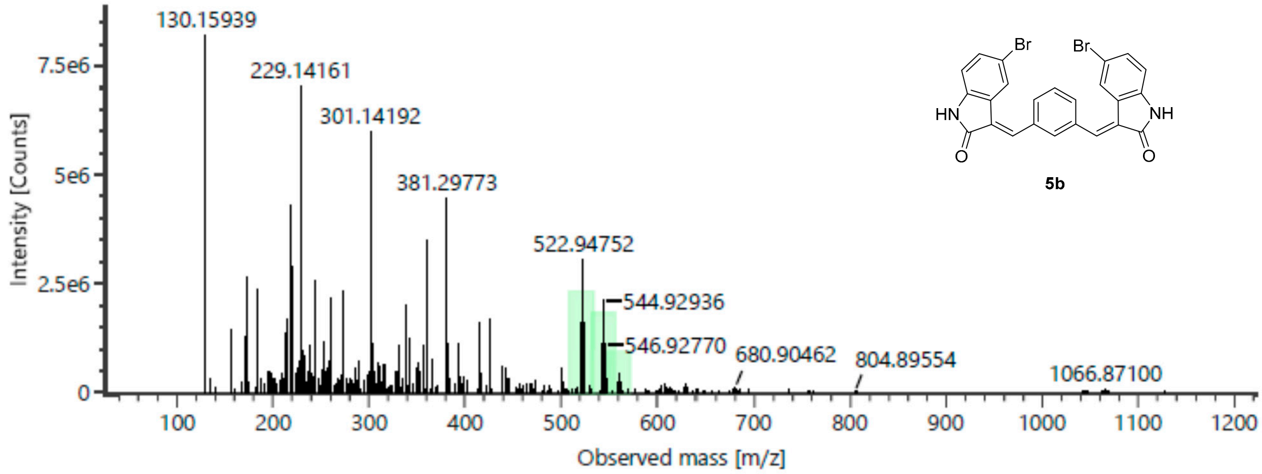
130.15939
229.14161
301.14192
381.29773
522.94752
544.92936
546.92770
680.90462
804.89554
1066.87100
7.5e6
5e6
2.5e6
0
Intensity [Counts]
Observed mass [m/z]
Br
HN
NH
O
5b

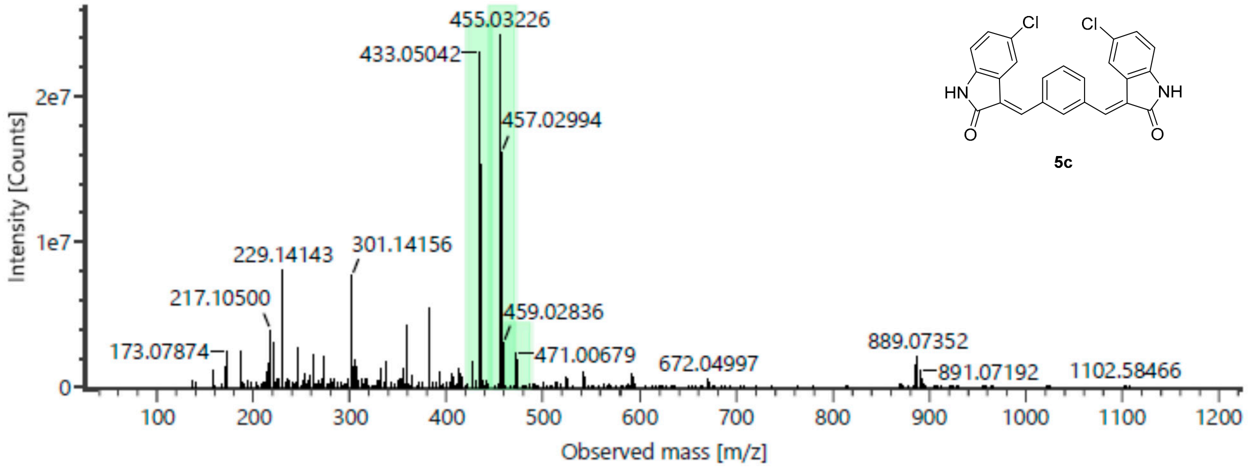
455.03226
433.05042
457.02994
229.14143
301.14156
217.10500
173.07874
459.02836
471.00679
672.04997
889.07352
891.07192
1102.58466
2e7
1e7
0
Intensity [Counts]
Observed mass [m/z]
Cl
HN
NH
O
5c

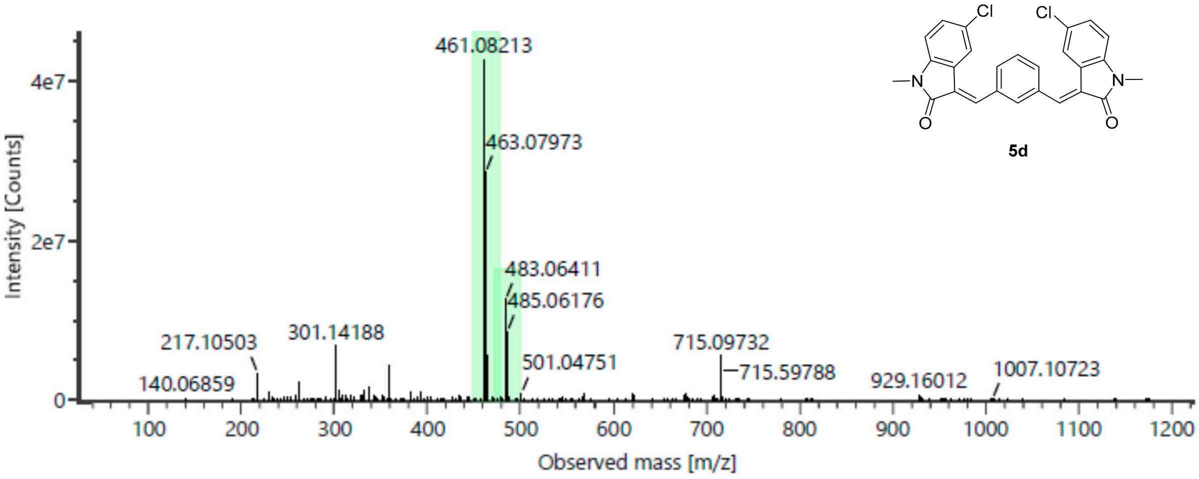
461.08213
463.07973
483.06411
485.06176
501.04751
217.10503
301.14188
140.06859
715.09732
715.59788
929.16012
1007.10723
Intensity [Counts]
Observed mass [m/z]
5d

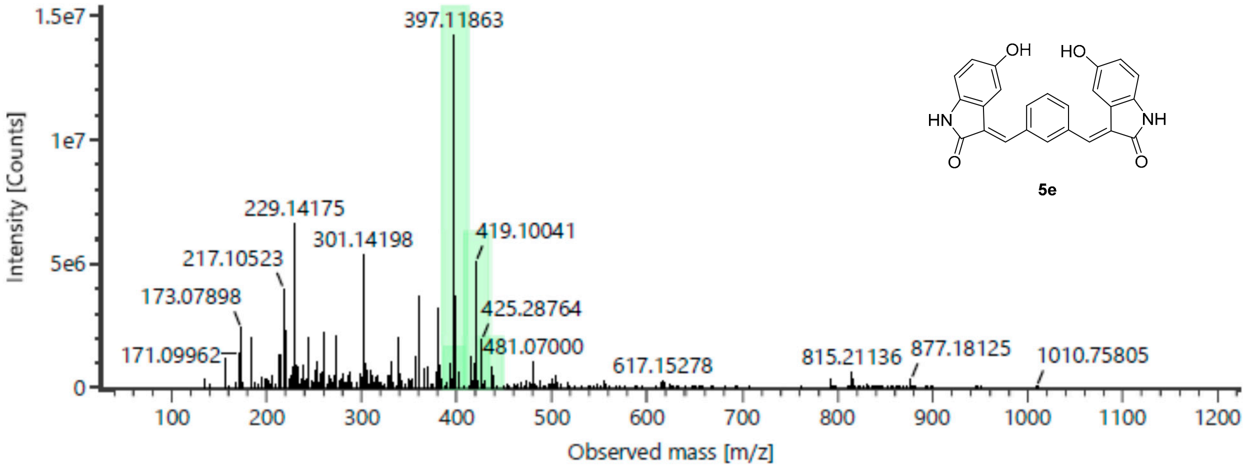
397.11863
229.14175
301.14198
217.10523
173.07898
171.09962
419.10041
425.28764
481.07000
617.15278
815.21136
877.18125
1010.75805
Intensity [Counts]
Observed mass [m/z]
5e

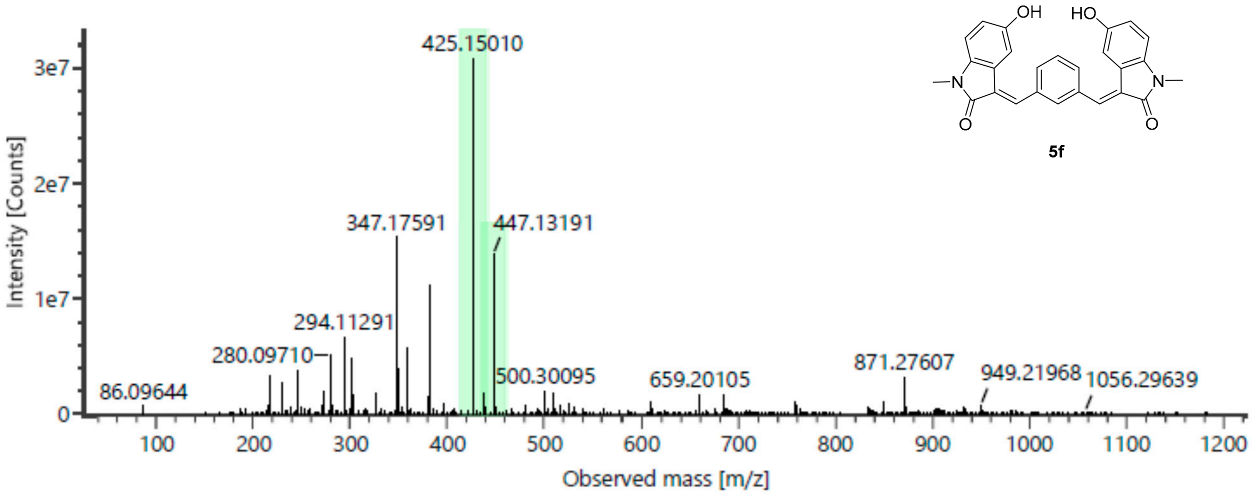
425.15010
347.17591
447.13191
294.11291
280.09710
86.09644
500.30095
659.20105
871.27607
949.21968
1056.29639
Intensity [Counts]
Observed mass [m/z]
5f

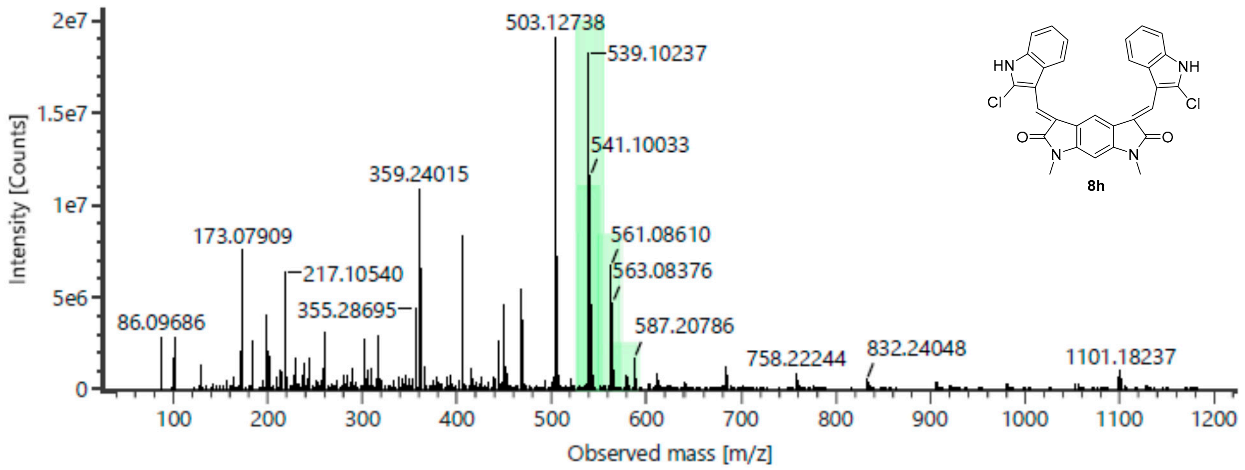

Intensity [Counts]
Observed mass [m/z]
503.12738
539.10237
541.10033
359.24015
173.07909
217.10540
355.28695
86.09686
561.08610
563.08376
587.20786
758.22244
832.24048
1101.18237
HN
NH
Cl
O
N
8h

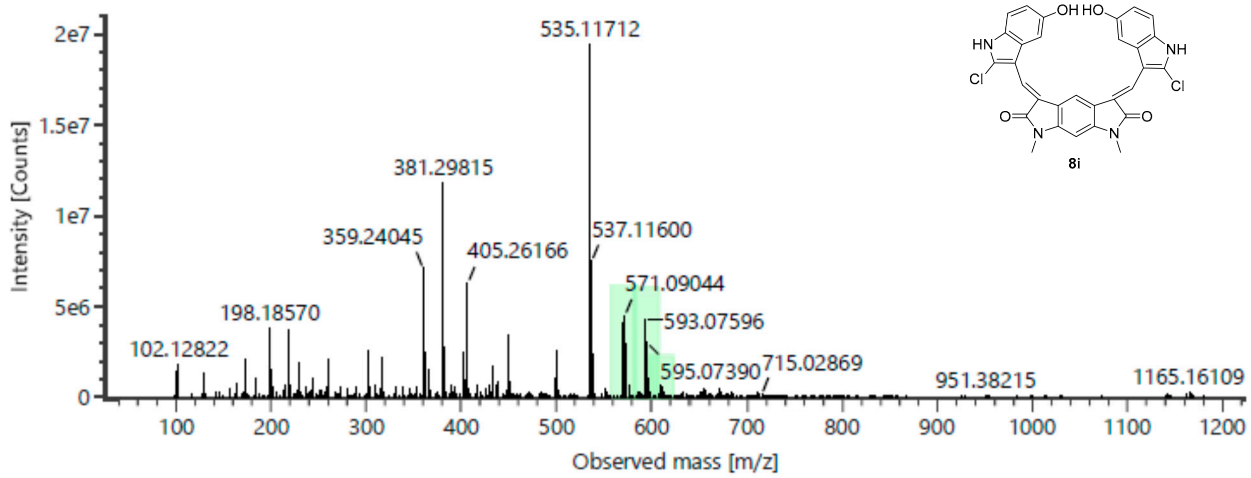

Intensity [Counts]
Observed mass [m/z]
535.11712
381.29815
359.24045
405.26166
537.11600
198.18570
102.12822
571.09044
593.07596
595.07390
715.02869
951.38215
1165.16109
OH HO
HN
NH
Cl
O
N
8i

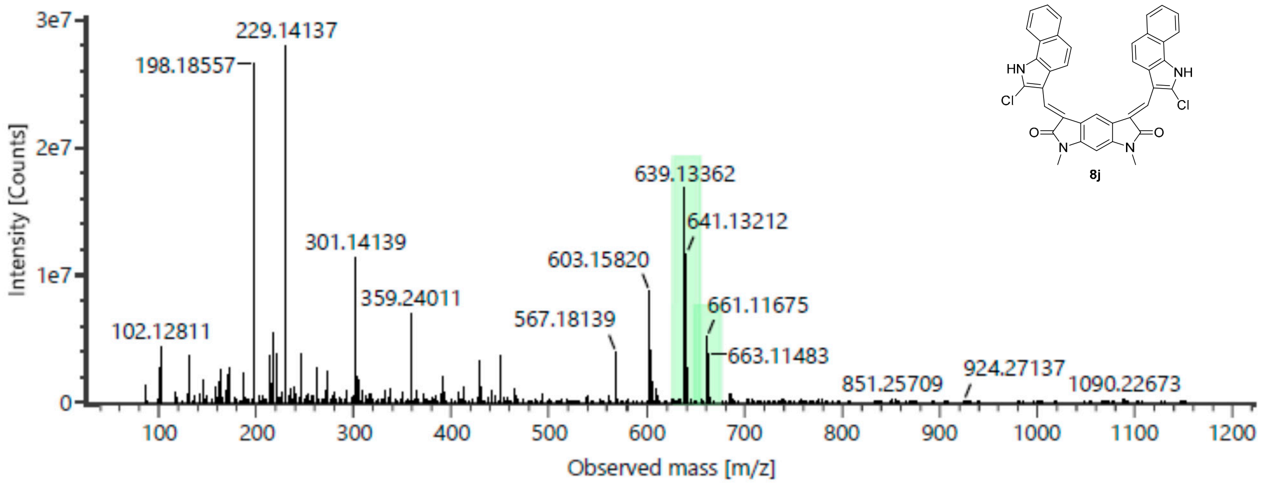

Intensity [Counts]
Observed mass [m/z]
229.14137
198.18557
301.14139
359.24011
102.12811
639.13362
641.13212
603.15820
567.18139
661.11675
663.11483
851.25709
924.27137
1090.22673
HN
NH
Cl
O
N
8j
